# Identification of TonB-dependent siderophore receptor inhibitors against *Flavobacterium columnare* using a structure-based high-throughput virtual screening method

**DOI:** 10.3389/fmicb.2024.1392178

**Published:** 2024-05-21

**Authors:** Minghao Li, Baipeng Chen, Ming Xu, Fulong Li, Yi Geng, Defang Chen, Ping Ouyang, Xiaoli Huang, Yongqiang Deng

**Affiliations:** ^1^Fisheries Research Institute, Sichuan Academy of Agricultural Sciences, Chengdu, Sichuan, China; ^2^Department of Aquaculture, College of Animal Science & Technology, Sichuan Agricultural University, Chengdu, Sichuan, China; ^3^Department of Basic Veterinary, College of Veterinary Medicine, Sichuan Agricultural University, Chengdu, Sichuan, China

**Keywords:** *Flavobacterium columnare*, TonB dependent siderophore receptor, virtual screening, exoxolone, prokaryotic transcriptome

## Abstract

TonB-dependent siderophore receptors play a critical transport role for *Flavobacterium columnare* virulence formation and growth, and have become valuable targets for the development of novel antimicrobial agents. Traditional Chinese medicine has demonstrated notable efficacy in the treatment of fish diseases and includes potential antibacterial agents. Herein, we performed molecular docking-based virtual screening to discover novel TonB-dependent siderophore receptor inhibitors from traditional Chinese medicine and provide information for developing novel antibacterial agents. Firstly, we efficiently obtained 11 potential inhibitors with desirable drug-like characteristics from thousands of compounds in the TCM library based on virtual screening and property prediction. The antibacterial activity of Enoxolone, along with its interaction characteristics, were determined via an MIC assay and molecular dynamic simulation. Transcriptional profiling, along with validation experiments, subsequently revealed that an insufficient uptake of iron ions by bacteria upon binding to the TonB-dependent siderophore receptors is the antibacterial mechanism of Enoxolone. Finally, Enoxolone's acceptable toxicity was illustrated through immersion experiments. In summary, we have used virtual screening techniques for the first time in the development of antimicrobial agents in aquaculture. Through this process, we have identified Enoxolone as a promising compound targeting the TonB-dependent siderophore receptor of *F. columnare*. In addition, our findings will provide new ideas for the advancement of innovative antimicrobial medications in aquaculture.

## 1 Introduction

*Flavobacterium columnare* is a common Gram-negative aerobic aquatic pathogen that is widespread in freshwater worldwide. *F. columnare* infects an extremely wide range of freshwater fish, including Cyprinidae, Siluridae, Percoidea, and Salmonidae, and is a pathogen affecting the global aquaculture industry (Staroscik and Nelson, 2008; Morley and Lewis, 2010; Verma et al., 2015; Jia et al., 2021; Kitiyodom et al., 2021; Ponpukdee et al., 2021; Wang et al., 2022). Currently, the optimal treatment for *F. columnare* is the use of antibiotics, chemicals, and vaccines (Cherian et al., 2023; Guo et al., 2023). However, due to the misuse of antibiotics, the ineffectiveness of chemical agents and the difficulties in promoting vaccines, there is still a lack of effective drug treatment in practice. Furthermore, with the growing limitations on the use of antibiotic-based antimicrobial agents, there is an escalating urgency to create novel antimicrobial agents.

Iron is an essential nutrient for bacterial growth and is involved in many important biological processes, including electron transfer, respiration, DNA synthesis, the tricarboxylic acid cycle, and biofilm formation (Taudte et al., 2016). In the natural water environment, free iron ions are mainly present in very small amounts in the form of trivalent iron ions. The TonB-dependent siderophore receptor-mediated iron transport pathway is thought to be the predominant mode of trivalent iron uptake by Gram-negative bacteria, including *F. columnare* (Guan et al., 2013). The transport process is powered by a system consisting of ExbB, ExbD, and the periplasmic protein TonB (Moeck and Coulton, 1998). Furthermore, due to its significance as a crucial element in the virulence of bacterial pathogens, the iron ion uptake system has been targeted for the creation of innovative antimicrobial agents. And key proteins within this system have been specifically focused on for this purpose (Lemos and Balado, 2020; Chan et al., 2023). Evidence has demonstrated that the TonB-dependent iron uptake system is required for the virulence process of *F. columnare* (Conrad et al., 2022). Therefore, it is appealing to focus on crucial proteins in this system to create innovative antimicrobial agents against *F. columnare*.

Compared to human drugs, aquatic drug development is more focused on achieving cheap costs and speed. Traditional drug development models are frequently unsuitable for aquatic drug development due to its high level of complexity and length. Therefore, the utilization of the molecular docking virtual screening technique shows great potential as a highly effective and appealing substitute in the realm of aquatic drug discovery (Rather et al., 2023). Compared to traditional drug development processes, virtual screening techniques can reduce development costs and speed up the process, making them ideal for researchers with limited resources to find potential drug candidates (Hernández-Silva et al., 2023). However, unfortunately, despite the mature applications in human drug discovery, virtual screening techniques have not been reported in the field of aquatic drug discovery.

Traditional Chinese medicine (TCM), which includes crude extracts and effective components derived from herbs, has been used in China for thousands of years. In recent decades, it has also been extensively utilized in fish aquaculture and has emerged as a favored therapy to substitute antibiotics and chemicals due to its minimal toxicity and potent curative impact (Zhu, 2020). Given the exorbitant expenses, lengthy duration, and inherent challenges associated with innovative drug research, TCM plays a clear and key role in the source of inspiration for drug discovery.

Therefore, in order to screen for a novel antibacterial agent against *F. columnare*, this study is the first to use virtual screening techniques for the development of aquatic drugs. Specifically, a library of TCM compounds was screened using the TonB-dependent siderophore receptor protein from *F. columnare* as the receptor. Further, the mechanism of bacterial inhibition and the biotoxicity of the lead compound, Enoxolone (also known as 18β-Glycyrrhetinic Acid), were also investigated. The results of this study will lay the foundation for the prevention and control of *F. columnare* and the development of related drugs.

## 2 Materials and methods

### 2.1 Structure preparation

Since the protein structure of the TonB-dependent siderophore receptor of *F. columnare* was not available in the PDB database, the SWISS-MODEL, Phyre2, Robetta, and ColabFold web servers were introduced to generate protein structural data (Kelley et al., 2015; Waterhouse et al., 2018; Baek et al., 2021; Mirdita et al., 2022). During the modeling process, all parameters were selected as default parameters. The predicted protein structure was modeled after the amino acid sequence of the TonB-dependent siderophore receptor in the reference genome of *F. columnare* in the NCBI database (WP_065213071.1) (Agarwala et al., 2017). To validate the model quality, the SAVES web server (Available online at https://servicesn.mbi.ucla.edu/SAVES/, accessed July 10, 2022) was used to analyze the stereochemistry of the obtained protein models and to assess the model quality. The model with the highest overall score was selected for molecular docking. The protein structure was processed with the Protein Preparation wizard in Schrodinger suite. Then, the AUTODOCK 4.0 MGL tool was used to convert the protein PDB files into the required PDBQT format.

The 3D structures of the molecular ligands used in this study were downloaded from the Traditional Chinese Medicine Library (Catalog No. L8300, Selleck Chemicals, Houston, TX, USA). Openbabel was used to split the downloaded SDF files and convert them to the PDB format (O'Boyle et al., 2011). Finally, the AUTODOCK 4.0 MGL tool was used to convert the file to the PDBQT format, assuming a neutral condition (pH = 7.4) to satisfy the requirements for bacterial growth.

### 2.2 The molecular docking-based virtual screening

We uploaded the protein 3D structure files to the CASTp 3.0 server to predict active pocket locations based on geometric and topological properties (Tian et al., 2018), and labeled amino acid residues to visualize pockets in MGLTools 1.5.6 (https://ccsb.scripps.edu/mgltools/). The position of the catalytic site was manually adjusted to completely wrap the pocket. The final parameters for the catalytic sites were set as center-x = 2.472, center-y = 9.250, and center-z = −1.556, with a grid size of 29 × 29 × 29. Autodock Vina (version 1.2.2) and R (version 4.2.3) were utilized to perform high-throughput molecular docking-based virtual screening (Trott and Olson, 2010). Throughout the docking, the ligand was in a flexible form, while the protein was in a rigid form. The maximum number of binding modes to output was fixed at 9, and the exhaustiveness level (controlling the number of independent runs performed) was set at 8. The conformation with the best affinity was selected as the final docking conformation. The molecular dynamics simulations were carried out with Desmond/Maestro non-commercial version 2022.1 as a molecular dynamics software to evaluate the stability and binding interactions of the complexes (Bowers et al., 2006). TIP3P water molecules were added to the systems, which were then neutralized by a 0.15 M NaCl solution. After the minimization and relaxation of the system, the production simulation was performed for 100 ns in an isothermal–isobaric ensemble at 300 K and 1 bar. The trajectory coordinates were recorded every 100 ps. The molecular dynamics analysis was performed using the Simulation Interaction Diagram from Desmond. Pymol 1.8.6 was used for the visualization of the complexes (Lashkov et al., 2021). Furthermore, the top 20 ranked compounds were selected for further property evaluation.

### 2.3 Compound property prediction

Online platforms were used to predict the drug properties, bioactivity score, ADME properties, and toxicity profiles of the compounds. Specifically, the ADMETlab 2.0 and SWISS ADME were used to predict the drug properties, ADME properties, and toxicity profile, while Molinspiration, a molecular property calculator based on Java (Available online at http://www.molinspiration.com/, accessed July 22, 2022), was used to predict the bioactivity scores (Daina et al., 2017; Xiong et al., 2021). Florfenicol, a commonly used aquatic antibiotic, was selected as a control to eliminate compounds with poor drug-like properties. The screened compounds participated in the subsequent MIC (minimum inhibitory concentration) assay.

### 2.4 Bacterial strains

The experimental strain of the pathogen *F. columnare* was isolated and purified from the gill tissue of dying diseased fish via inoculation with Cytophaga agar containing 0.5 g/L Tryptone, 0.2 g/L Beef extract, 0.5 g/L Yeast extract, 0.2 g/L Na-acetate, and 1% Agar. The strain was maintained as a frozen stock at −80°C in 50% (*v*/v) glycerol at Sichuan Agricultural University. After resuscitating the frozen stock culture, a single colony was transferred from the Cytophaga agar to 10 mL of *Flavobacterium columnare* growth media (FCGM, containing 8.0 g/L Tryptone, 0.8 g/L Yeast extract, 1.0 g/L MgSO4·7H_2_O, 0.74 g/L CaCl_2_·2H_2_O, 5.0 g/L NaCl, 1.5 g/L Sodium Citrate) broth by picking it up with an inoculating loop and cultured at 28°C with shaking at 200 rpm for 24 h. The strain was identified as *F. columnare* through DNA extraction, PCR amplification of the 16SrDNA fragment (forward primer: 5′-AGAGTTTGATCCTGGCTCAG-3′; reverse primer: 5′-GGTTACCTGTTACGGACTT-3′), and a BLASTN comparison of the NCBI database (https://blast.ncbi.nlm.nih.gov/Blast.cgi).

### 2.5 MIC determination

A determination of the MIC of different small-molecule compounds against *F. columnare* was carried out in 96-well U-bottom polystyrene microtiter plate using the broth dilution method. All compounds were purchased from Selleck Chemicals and dissolved in dimethyl sulfoxide (DMSO) to prepare a 10 mM master batch. The 2-fold serial dilution of each compound ranging from 128 to 0.0625 μg mL^−1^ was used for the assay (the maximum concentration of Uvaol, which is extremely insoluble in water, is 32 μg mL^−1^). The FCGM broth (pH = 7.4) was added to all wells as the medium and 0.5 McFarland bacteria were cultured for 24 h (Farmer, 2004). The bacterial growth was observed by using 0.015% Resazurin dye to examine the viability of the bacterial cells. FCGM broth supplemented with DMSO and cultures without the compound addition were used as the negative and positive controls, respectively. Meanwhile, the MIC of Florfenicol was tested in the same way against *F. columnare*.

### 2.6 RNA sequencing and analysis

#### 2.6.1 Extraction and detection of total RNA

Overnight cultures of *F. columnare* were inoculated into 10 mL of fresh FCGM broth and cultured at 28°C with shaking. When the OD_600_ reached 0.5, DMSO-solubilized Enoxolone (1 × MIC: 8 μg/mL) was added to the medium. After 2 h of incubation, the bacterial cells were collected via cryogenic centrifugation and stored at −80°C until further use. Additionally, bacterial cells treated with equal amounts of DMSO were used as controls. Three biological replicates were carried out for both the control and the experimental groups. Total RNA was extracted from the bacterial cells using the TRIzol^®^ Reagent according to the manufacturer's instructions (Invitrogen), and genomic DNA was removed using DNase I (TaKara, Kusatsu, Japan). Then, the RNA quality was determined with a 2100 Bioanalyzer (Agilent) and quantified using the ND-2000 (NanoDrop Technologies, Wilmington, DE, USA). Then, 1% agarose gel electrophoresis was used to detect the integrity of the total RNA. Only high-quality RNA samples (OD260/280 ≥ 1.8, OD260/230 ≥ 1.0, RIN ≥ 6.5, 28S:18S ≥ 1.0, ≥50 ng/μL, ≥ 1 μg) were utilized for the subsequent construction of the library.

#### 2.6.2 cDNA library construction and transcriptome sequencing

The RNA-seq transcriptome library was prepared following the TruSeqTM RNA sample preparation Kit from Illumina (San Diego, CA, USA) using 2 μg of total RNA. A Ribo-zero Magnetic kit (Epicentre, Madison, WI, USA) was used to remove rRNA and purify mRNA. The mRNA was randomly broken into small fragments of about 200 bp and used as a template. Double-stranded cDNA was synthesized using a SuperScript double-stranded cDNA synthesis kit (Invitrogen, Carlsbad, CA, USA) with random hexamer primers (Illumina). The synthesized double-stranded cDNA was added to the End Repair Mix to complement its flat end. The 5′ end was phosphorylated, and an A base was added to the 3′ end to connect it to the Y-shaped sequencing connector. Libraries were size-selected for cDNA target fragments of 200 bp on 2% Low Range Ultra Agarose followed by PCR amplification using Phusion DNA polymerase (NEB) for 15 PCR cycles. The quality and number of cDNA libraries were evaluated using the TBS380 (Invitrogen, Carlsbad, CA, USA). Finally, the paired-end RNA-seq sequencing library was sequenced with the Illumina Novaseq (2 × 150 bp read length).

#### 2.6.3 Bioinformatics analysis

Clean reads were selected by removing low-quality sequences, reads with more than 5% N bases (unknown bases), and reads containing adaptor sequences using a Perl program. The genome of *Flavobacterium columnare* strain F2S17 (NZ_CP054494) in the NCBI database was selected as the reference genome. Bowtie2 (http://bowtie-bio.sourceforge.net/bowtie2/index.shtml) was used to compare the high quality of the sample reads against the reference genome. Using the blast method, 10,000 raw reads were randomly selected from each sample and compared with the Rfam database. The percentage of rRNA in each sample was calculated based on the annotation results to assess the rRNA contamination rate. Gene expression was calculated using RSEM (http://deweylab.github.io/RSEM/) with a maximum-likelihood abundance estimation model based on a maximum expectation algorithm, and expression levels were measured using FPKM and TPM. A statistical analysis of differentially expressed genes (DEGs) was performed using the edgeR software package (http://www.bioconductor.org/packages/2.12/bioc/html/edgeR.html) with *p*-value < 0.05 and |log2FC| ≥ 1 as the default screening criteria. In addition, a functional enrichment analysis of the screened DEGs was performed using Goatools (https://github.com/tanghaibao/GOatools) and KOBAS (http://kobas.cbi.pku.edu.cn) to determine the extent of DEG enrichment in different GO terms and KEGG pathways.

### 2.7 Cell viability assays

*F. columnare* strains were streaked from freezer stocks onto Cytophaga agar and incubated at 28°C for 24 h. Growth from the Cytophaga agar was then used to inoculate 10 mL of FCGM broth, which was incubated at 28°C with rotation to 0.5 McFarland. Enoxolone was added to the medium to a final MIC (8 μg/mL). For some experiments, ferric chloride (FeCl_3_) and ferrous sulfate (FeSO_4_) were added to 10 mL of FCGM cultures containing Enoxolone, respectively. Ferric chloride and ferrous sulfate were added in increments of 50 μM or 100 μM. Cultures without Enoxolone addition were used as controls. All cultures were set up in three parallels and incubated for 24 h at 28°C. We added 100 μL of the incubated culture to a 96-well plate. The cell viability was measured in triplicate using CCK-8 (Beyotime Biotechnology, Shanghai, China) at a dose of 10 μL per well^−1^. The absorbance values were measured at wavelength of 450 nm using an ultraviolet–visible spectrophotometer according to the manufacturer's protocol.

### 2.8 Biotoxicity determination

Sixty healthy largemouth bass (0.99 ± 0.31 g) were purchased from a farm in Sichuan. They were acclimated in the laboratory for 2 weeks prior to the experiment. All largemouth bass were divided into 6 groups (1 control and 5 experimental groups), each of which contained 10 largemouth bass. The control group received DMSO immersion (1%), while the experimental group was immersed in aeration water containing 4 μg/mL, 8 μg/mL, 16 μg/mL, 32 μg/mL, and 64 μg/mL of Enoxolone, respectively. During the experiment, the largemouth bass were exposed to an uninterrupted oxygen supply, the pH was marinated at 6.5–8.0, and water temperature was about 25°C. The experimental period was set to 96 h, and the number of deaths and clinical symptoms of the largemouth bass were recorded daily.

### 2.9 Statistical analysis

Data on the cell viability were analyzed and expressed as mean ± standard deviation. A statistical analysis was carried out using an analysis of variance (ANOVA) followed by multiple comparison tests (LSDs). If the variance among groups was not homogenous, Dunnett's T3 test was performed. A value of *p* < 0.05 was considered statistically significant and labeled with an asterisk (^*^). GraphPad Prism 8 was used for the graphical representations.

## 3 Results

### 3.1 Homology modeling and model evaluation

The TonB-dependent siderophore receptor protein lacks PDB entry; for this reason, the structure of the protein has been depicted through homology modeling. Amongst the 701 amino acids of TonB-dependent siderophore receptors, 5.85%, 49.50%, and 44.65% of these amino acids construct α-helices, β-strands, and transmembrane helices, respectively. SWISS-MODEL, Phyre2, Robetta, and ColabFold predicted the amino acid data of the proteins to generate structural images. Supplementary Figure S1 shows the 3D conformation of the *F. columnare* TonB-dependent siderophore receptor proteins constructed using different procedures. All protein models have a similar structure, namely a β-barrel structure formed of multiple β-folds and a plug structure contained in its center. The reasonableness of all modeled target protein topologies was analyzed using SAVES web servers. A Ramachandran plot was used to visualize whether the dihedral angles of the amino acid residues in the homologous modeled protein structures were in reasonable regions. A more rational protein structure has more amino acid residues in the best regions. According to the Ramachandran plot, the percentages of amino acid residues in the best regions for the SWISS-MODEL, Phyre2, Robetta, and ColabFold rank1 models were 84.5%, 77.9%, 88.5%, and 86.6%, respectively. In addition, non-bonded interactions between different atom types were analyzed in the ERRAT program (Colovos and Yeates, 1993). As an important indicator for assessing the three-dimensional structure of proteins via crystallography, the high factors obtained in ERRAT represent the high resolution of the structure. The results showed that the ColabFold rank1 model protein used for the analysis received the highest score of 83.847, followed by SWISS-MODEL (81.719), Robetta (81.584), and Phyre2 (49.461) (Supplementary Figures S2–S5). The PAE (prediction alignment error) results indicated that the ColabFold rank1 model protein has more confidence in the expected positions of amino acid residues in the five 3D structures generated by ColabFold, indicating that the ColabFold rank1 model protein has the highest confidence (Supplementary Figure S2D). In combination with the above data, the ColabFold rank1 model protein was used as the receptor protein for the subsequent screening.

### 3.2 Molecular docking based virtual screening

**Figure 1:**
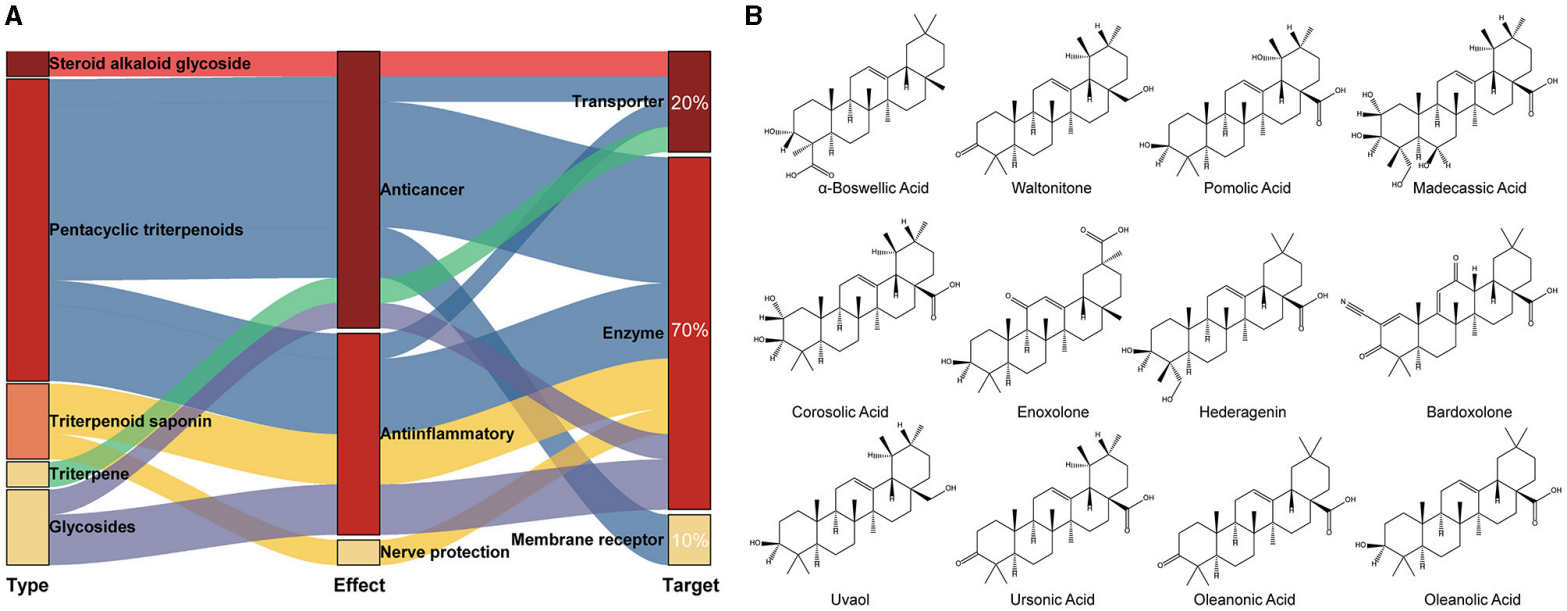
Classification of the top 20 scoring compounds. **(A)** Sankey diagram of tightly bound compounds in the TCM library; **(B)** the 2D structure of pentacyclic triterpenes among the top 20 compounds with highest scores.

The crystal structure of the target protein was not resolved. Meanwhile, the target protein has low identity with the protein in the PDB database and is highly conserved in the genus (Supplementary Figure S6). Therefore, CASTp was used to characterize and measure the geometric and topological properties of protein structures to complete the visualization of TonB-dependent siderophore receptor protein pocket channels. The results of the CASTp calculation are shown in Supplementary Figure S7. After localizing the active pocket, AutoDock Vina was used to dock the unique conformation of a total of 1,335 TCM ligands to the active pocket in the TonB-dependent siderophore receptor. The docking scores of each protein–ligand complex generated via molecular docking were ranked to screen for stably bound ligands. As revealed in Table 1, the top 20 screened hits were listed according to their docking scores. In addition to the highest docking score that Solamarine (CAS: 20311-51-7) showed in terms of its docking with the receptor, all other compounds had docking scores above −10.9 kcal/mol. To more fully cognize the similarities and differences among the top 20 screens, compounds were searched by type, effect, and target of action to create a Sankey diagram. The results showed that the top 20 compounds were mostly pentacyclic triterpenoids with similar structures (linked by six isoprene units) (Figure 1). Most of the compounds have anticancer and anti-inflammatory effects, with the most frequent targets being enzymes (14; 70%), followed by transporters (four; 20%) and membrane receptors (two; 10%).

**Table 1:** Results of molecular docking analysis of compounds in TCM libraries by Autodock Vina.

**Rank**	**Ligand name**	**CAS Number**	**Mol Formula**	**Affinity (kcal/mol)**
1	Solamargine	20311-51-7	C45H73NO15	−12.8
2	Oleanonic Acid	17990-42-0	C30H46O3	−12.1
3	Madecassic acid	18449-41-7	C30H48O6	−12
4	Corosolic acid	4547-24-4	C30H48O4	−11.9
5	Pomolic acid	13849-91-7	C30H48O4	−11.7
6	Asiaticoside	16830-15-2	C48H78O19	−11.7
7	Obacunone	751-03-1	C26H30O7	−11.7
8	Tenuifolin	20183-47-5	C18H16O4	−11.7
9	α-Boswellic acid	471-66-9	C30H48O3	−11.5
10	Waltonitone	1252676-55-3	C30H48O2	−11.5
11	Hederagenin	465-99-6	C30H48O4	−11.4
12	Uvaol	545-46-0	C30H50O2	−11.3
13	Oleanolic acid	508-02-1	C30H48O3	−11.1
14	Ursonic acid	6246-46-4	C30H46O3	−11.1
15	Enoxolone	471-53-4	C30H46O4	−11
16	Pseudoprotodioscin	102115-79-7	C51H82O21	−11
17	Bardoxolone	218600-44-3	C31H41NO4	−10.9
18	Protodioscin	55056-80-9	C51H84O22	−10.9
19	Dioscin	19057-60-4	C45H72O16	−10.9
20	Saikosaponin A	20736-09-8	C42H68O13	−10.9

### 3.3 Drug-Likeness and biological activity of screening hits

Excellent drug-likeness and biological activity contribute to reducing the risk of failure of drug candidates in the production application stage. The ADME/T and biological activity of the top 20 ranked compounds obtained from the virtual screening were predicted and further filtered using Florfenicol as a standard. The results show that the 14 compounds conform to Lipinski's rule and have relatively good characteristic parameters (Table 2). In addition, most compounds do not cross the blood–brain barrier and do not inhibit CYP family enzymes, showing a high safety profile (Table 3). The Molinspiration server was used to calculate the bioactivity scores of the compounds on GPCRs (G protein-coupled receptors), nuclear receptors, ion channels, kinases, proteases, and other enzymes. The results showed that all the compounds screened had more than moderate activity (−5 to 0) against the predicted targets, with some showing significant activity (score > 0) (Table 4). The results of the toxicity prediction show that some of the compounds have potential cardiotoxicity, carcinogenicity, mutagenicity, and aquatic toxicity (Table 5). Since the compounds will be used in an aqueous environment, Lipinski's rule, the blood–brain barrier, CYP family enzymes, and toxicity (hERG, AMES Toxicity Carcinogenicity, and LC50FM) were set as screening conditions in this study to make the compounds bioenvironmentally friendly. Ultimately, Solamargine, Asiaticoside, Obacunone, Tenuifolin, Pseudoprotodioscin, Bardoxolone, Protodioscin, Dioscin, and Saikosaponin A were removed due to ineligible drug-likeness, and the remaining compounds were used in subsequent assays for antimicrobial activity.

**Table 2:** Drug-likeness physicochemical properties of compounds.

**Compound**	**Mwt (g/mol)^a^**	**logP^b^**	**TPSA^c^**	**nHA^d^**	**nHD^e^**	**nRot^f^**	**Lipinski Rule^g^**
Solamargine	867.5	2.97	238.48	16	9	7	Rejected
Oleanonic acid	454.34	6.35	54.37	3	1	1	Accepted
Madecassic acid	504.35	3.504	118.22	6	5	2	Accepted
Corosolic acid	472.36	5.541	77.76	4	3	1	Accepted
Pomolic acid	472.36	5.258	77.76	4	3	1	Accepted
Asiaticoside	958.51	2.156	315.21	19	12	10	Rejected
Obacunone	454.2	3.543	95.34	7	0	1	Accepted
Tenuifolin	296.1	3.524	47.92	4	1	2	Accepted
α-Boswellic acid	456.36	6.206	57.53	3	2	1	Accepted
Waltonitone	440.37	6.301	37.3	2	1	1	Accepted
Hederagenin	472.36	5.096	77.76	4	3	2	Accepted
Uvaol	442.38	6.621	40.46	2	2	1	Accepted
Oleanolic Acid	456.36	6.645	57.53	3	2	1	Accepted
Ursonic acid	454.34	6.103	54.37	3	1	1	Accepted
Enoxolone	470.34	5.627	74.6	4	2	1	Accepted
Pseudoprotodioscin	1,030.53	2.103	325.83	21	12	14	Rejected
Bardoxolone	491.3	4.646	95.23	5	1	1	Accepted
Protodioscin	1,048.55	1.649	346.06	22	13	14	Rejected
Dioscin	868.48	3.232	235.68	16	8	7	Rejected
Saikosaponin A	766.45	2.091	207.99	13	8	6	Rejected
Florfenicol	358.21	0.496	83.47	5	2	7	Accepted

^a^Molecular weight containing hydrogen atoms; ^b^oil-water partition coefficient; ^c^Total polar surface area and molecular volume; ^d^Number of hydrogen bond acceptors; ^e^Number of hydrogen bond donors; ^f^Number of rotatable bonds; ^g^MW ≤ 500; logP ≤ 5; Hacc ≤ 10; Hdon ≤ 5. If two properties are out of range, a poor absorption or permeability is possible, one is acceptable.

**Table 3:** ADME properties of compounds.

**Compound**	**GI absorption^a^**	**Caco-2 Permeability**	**BBB^b^**	**CYP1A2 inhibitor**	**CYP2C19 inhibitor**	**CYP2D6 inhibitor**	**CYP3A4 inhibitor**
Solamargine	Yes	−6.044	No	No	No	No	No
Oleanonic acid	No	−5.404	No	No	No	No	No
Madecassic acid	No	−5.757	No	No	No	No	No
Corosolic acid	No	−5.448	No	No	No	No	No
Pomolic acid	No	−5.422	No	No	No	No	No
Asiaticoside	Yes	−6.388	No	No	No	No	No
Obacunone	No	−5.316	Yes	No	No	No	No
Tenuifolin	No	−4.754	Yes	Yes	Yes	Yes	Yes
α-Boswellic acid	No	−5.297	No	No	No	No	No
Waltonitone	No	−5.136	No	No	No	No	No
Hederagenin	No	−5.407	No	No	No	No	No
Uvaol	No	−5.006	No	No	No	No	No
Oleanolic Acid	No	−5.37	No	No	No	No	No
Ursonic acid	No	−5.453	No	No	No	No	No
Enoxolone	No	−5.498	No	No	No	No	No
Pseudoprotodioscin	Yes	−6.212	No	No	No	No	No
Bardoxolone	No	−5.386	No	No	No	No	No
Protodioscin	Yes	−6.377	No	No	No	No	No
Dioscin	Yes	−5.978	No	No	No	No	No
Saikosaponin A	Yes	−5.589	No	No	No	No	No
Florfenicol	Yes	−5.94	No	No	No	No	Yes

^a^Gastrointestinal absorption; ^b^Blood-brain barrier.

**Table 4:** Bioactivity score of compounds.

**Compound**	**GPCR ligand**	**Ion channel modulator**	**Kinase inhibitor**	**Nuclear receptor ligand**	**Protease inhibitor**	**Enzyme inhibitor**
Solamargine	−2.45	−3.51	−3.52	−3.22	−1.92	−2.59
Oleanonic Acid	0.17	−0.14	−0.58	0.72	0.05	0.57
Madecassic acid	0.25	−0.1	−0.45	0.93	0.29	0.75
Corosolic acid	0.24	−0.13	−0.5	0.93	0.28	0.66
Pomolic acid	0.29	0.07	−0.38	0.84	0.18	0.72
Asiaticoside	−3.38	−3.7	−3.7	−3.55	−2.96	−3.26
Obacunone	0.15	−0.05	−0.57	0.6	0.07	0.41
Tenuifolin	0.13	−0.04	0.01	0.16	−0.17	0.18
α-Boswellic acid	0.24	−0.01	−0.35	0.67	0.25	0.58
Waltonitone	0.09	−0.2	−0.59	0.77	0.08	0.5
Hederagenin	0.23	−0.11	−0.37	0.76	0.16	0.66
Uvaol	0.2	−0.1	−0.39	0.83	0.19	0.58
Oleanolic Acid	0.28	−0.06	−0.4	0.77	0.15	0.65
Ursonic acid	0.18	−0.11	−0.68	0.84	0.13	0.61
Enoxolone	0.24	−0.09	−0.56	−0.79	0.21	0.7
Pseudoprotodioscin	−3.67	−3.78	−3.82	−3.75	−3.59	−3.63
Bardoxolone	−0.01	−0.2	−0.68	0.6	0.01	0.57
Protodioscin	−3.67	−3.79	−3.82	−3.75	−3.6	−3.62
Dioscin	−2.54	−3.5	−3.5	−3.11	−1.95	−2.58
Saikosaponin A	−1	−2.1	−1.88	−1.51	−0.68	−1.13
Florfenicol	0.14	−0.25	−0.08	0.21	0.47	0.47

**Table 5:** Toxicity Profile of compounds.

**Compound**	**hERG^a^**	**Carcinogencity**	**AMES Toxicity**	**LC_50_FM^b^**
Solamargine	Yes	No	No	5.084
Oleanonic Acid	No	No	No	5.931
Madecassic acid	No	No	No	5.53
Corosolic acid	No	No	No	6.509
Pomolic acid	No	No	No	6.677
Asiaticoside	No	No	No	6.647
Obacunone	No	No	No	6.677
Tenuifolin	No	Yes	Yes	5.188
α-Boswellic acid	No	No	No	6.805
Waltonitone	No	No	No	6.119
Hederagenin	No	No	No	6.11
Uvaol	No	No	No	6.575
Oleanolic Acid	No	No	No	6.42
Ursonic acid	No	No	No	5.911
Enoxolone	No	No	No	5.785
Pseudoprotodioscin	Yes	No	No	4.506
Bardoxolone	No	Yes	No	5.347
Protodioscin	Yes	No	No	3.823
Dioscin	Yes	No	No	5.334
Saikosaponin A	No	No	No	6.666
Florfenicol	No	Yes	Yes	3.508

^a^Human ether-a-go-go-related gene (hERG) blocker activity (inhibition of potassium channels leading to arrhythmias); ^b^Predicted LC_50_ for fathead minnow in 96 h (Units are -log10 [(mg/L)/(1000^*^MW)]).

### 3.4 *In vitro* antibacterial activity assay

**Figure 2:**
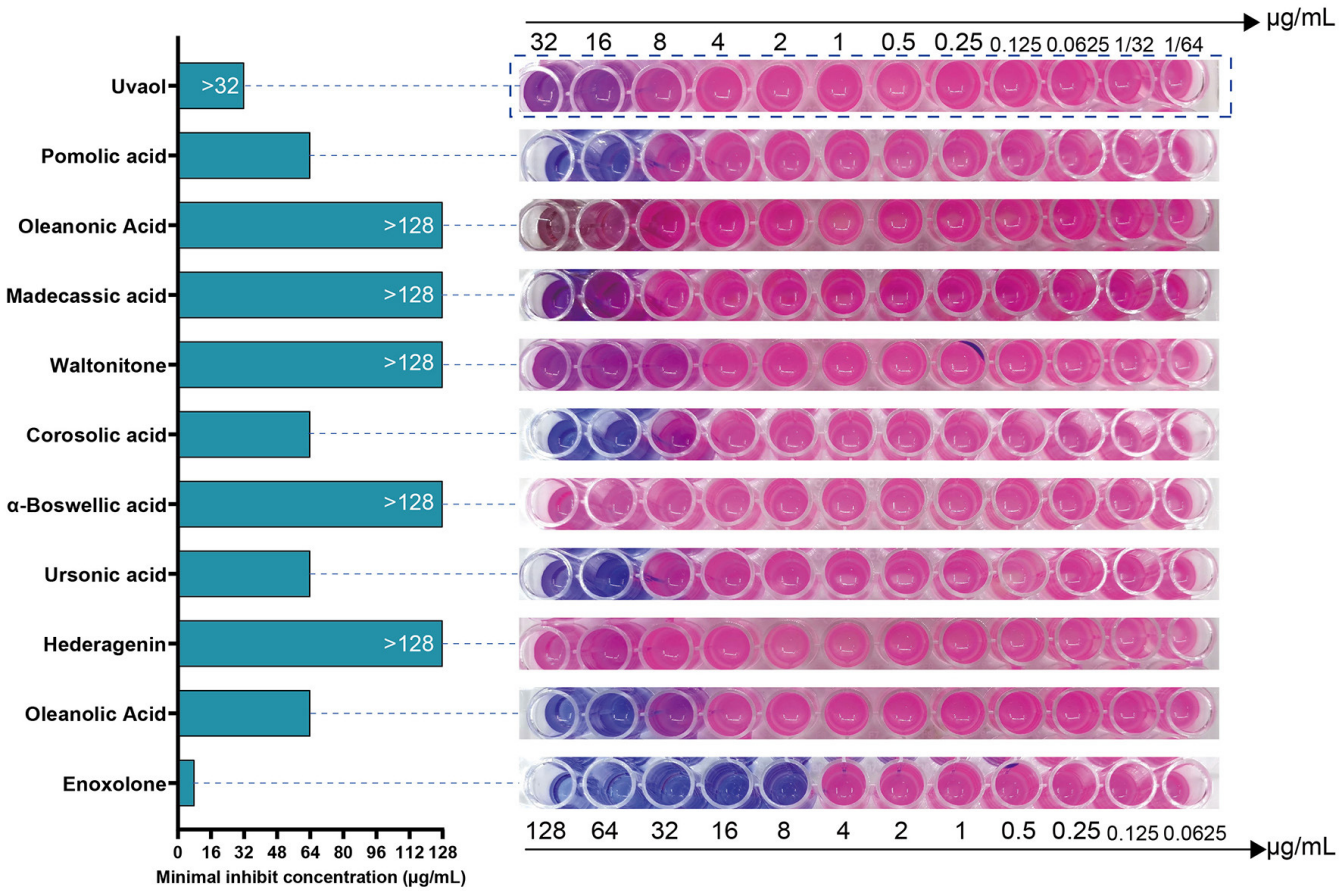
Resazurin based microdilution assay for MIC determination of compounds against *Flavobacterium columnare*. The MIC values of compounds against the *Flavobacterium columnare* are indicated in the left hand side. The growth of bacterial was observed by conversion of blue colored resazurin dye to pink one by viable bacterial cells.

We tested the antibacterial activity of the primary screened small-molecule compounds against *F. columnare* using a microdilution method based on Resazurin staining. The results showed that *F. columnare* showed strong resistance to Hederagenin, α-Boswellic acid, Waltonitone, Madecassic acid, Oleanonic Acid, and Uvaol. In contrast, Enoxolone, Oleanolic acid, Ursonic acid, Corosolic acid, and Pomolic acid showed antibacterial activity. Among all compounds, Enoxolone had the smallest MIC, which was 8 μg/mL (Figure 2). Meanwhile, the MIC of Florfenicol, the control drug, was determined to be 2 μg/mL. Therefore, Enoxolone was selected as a potential lead compound for subsequent experiments.

### 3.5 Molecular dynamic

**Figure 3:**
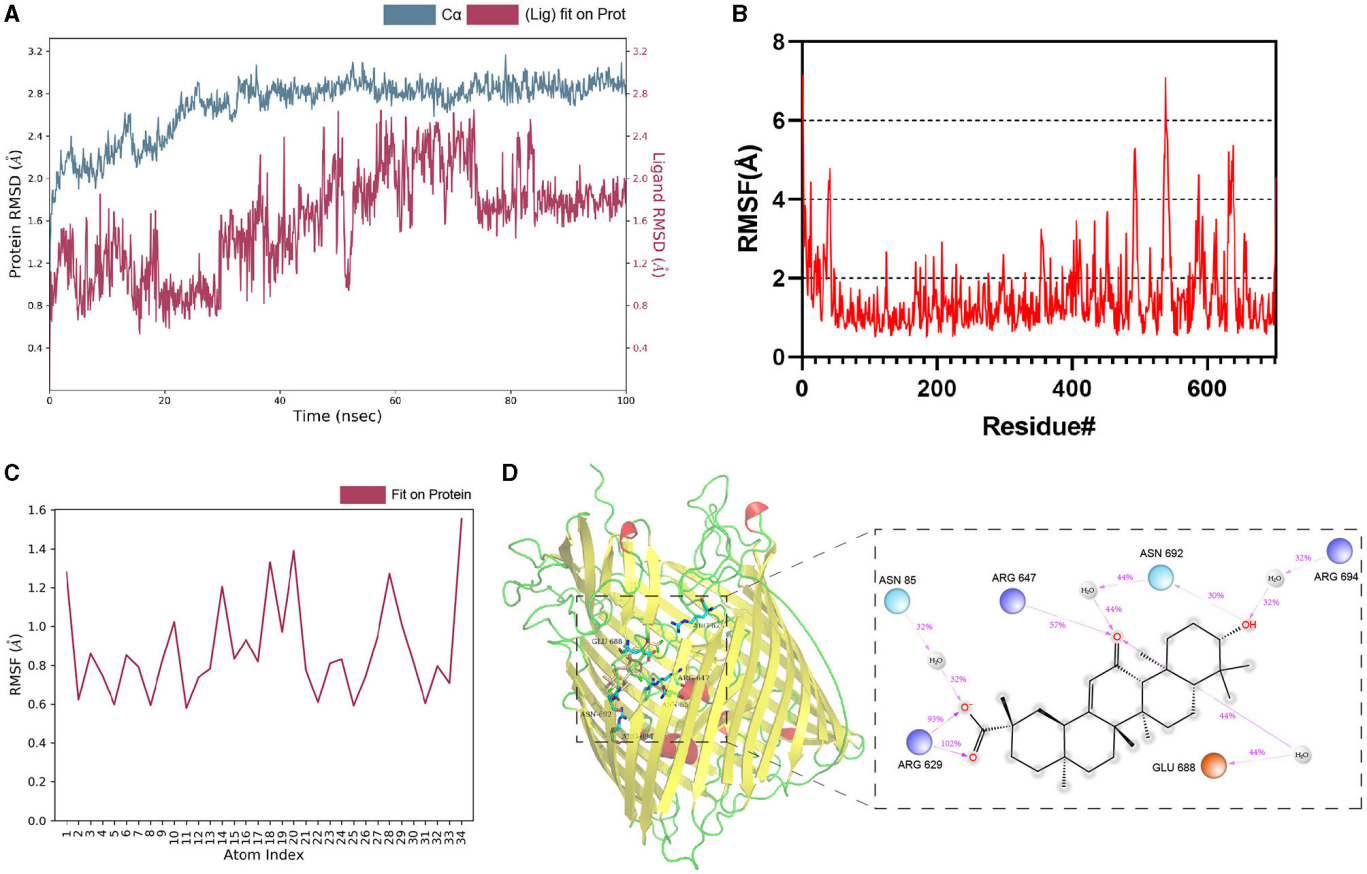
Molecular dynamics simulations of protein–Enoxolone complex. **(A)** RMSD values of backbone atoms of the protein and the ligand. **(B)** RMSFs of TonB-dependent siderophore receptor. **(C)** RMSFs of the heavy atoms in the ligand. **(D)** Interaction analysis of the complex. Interactions that occur for more than 30.0% of the simulation time in the selected trajectory are shown. It is possible to have interactions with >100%, as some residues may have multiple interactions of a single type with the same ligand atom. Dark-blue, light-blue, and orange circles represent positively charged, negatively charged, and polar amino acid residues, respectively.

The dynamic stability and interactions of the TonB-dependent siderophore receptor–Enoxolone complex were further evaluated with molecular dynamics simulations. To study the dynamic stability of the complex, root mean square deviations (RMSDs) from the starting structures were analyzed. The plots showed that the protein and ligand were in a steady state during the simulations. After equilibration, the RMSD of the protein stabilized at 2.6 Å to 2.8 Å, whereas the RMSD of the ligand stabilized at 1.6 Å to 2.0 Å (Figure 3A). Then, the overall calculated root mean square fluctuations (RMSFs) of the protein and ligand were also small (Figures 3B, C). These small fluctuations indicate the high stability of the initial conformation of the complex. In addition, the RMSD and RMSF between apo-protein and protein-ligand complex were also nearly (Supplementary Figure S8). After docking, ARG647, SER675, GLU688, and TRP693 bind to the ligand via hydrogen bonding. During the simulation, the protein and small molecules formed multiple sets of interactions, including hydrogen bonds, hydrophobic interactions, and water bridges (Supplementary Figure S9). ASN 85, ARG 629, ARG 647, GLU 688, ASN 692, and ARG 694 were recognized as key amino acid residues in the binding process. In particular, the hydrogen bond formed by ARG 629 with small molecules was believed to play a crucial role (Figure 3D). Therefore, Enoxolone displayed a high binding affinity for TonB-dependent siderophore receptors in this conformation.

### 3.6 RNA-seq quality assessment and determination of DEGs

To further explore the potential mechanism of Enoxolone inhibition in *F. columnare*, six prokaryotic transcriptome sequencing libraries were constructed, containing three DMSO-treated controls and three Enoxolone-treated groups. The six libraries were sequenced, and the data are summarized in Table 6. An average of 25,720,127 raw reads were obtained per sample, with Q20 and Q30 both over 93%. The percentage of rRNA assessed against the reference genome was < 10% for all samples. In addition, the sequenced sequences were evenly distributed across the genes, indicating unbiased sequencing (Supplementary Figure S10). The quality control data showed that the sequencing results met the requirements for prokaryotic transcriptome analysis. Further, all unigenes obtained in the assembly were blasted against six databases (NR, Swiss-Prot, Pfam, COG, GO, and KEGG), among which 2,715 genes were annotated (Supplementary Figure S11). The expression levels of the genes were normalized to FPKM and TPM. Prior to the DEG analysis, a principal component analysis (PCA) was performed based on the expression matrix. The results showed that there was a significant difference in gene expression between the different groups (Supplementary Figure S12A). DEGs were analyzed based on the read counts, and 76 DEGs (up: 52; down: 24) were identified using edgeR (*p*-value < 0.05 and |log2FC| ≥ 1 in normalized expression values) (Supplementary Figure S12B).

**Table 6:** Quality control data of the prokaryotic transcriptome.

**Sample name**	**Raw reads**	**Clean reads**	**Clean error rate (%)**	Clean Q20 (%)^*^	Clean Q30 (%)^**^	**rRNA ratio (%) of reference**	**Uniq mapped reads ratio (%)**
E3	23301460	23079410	0.0248	98.12	94.25	2.03	88.54
E2	31012756	30541910	0.0253	97.9	93.8	1.67	88.69
E1	24479704	24205768	0.025	98.06	94.17	2.01	88.77
D3	26274002	26162182	0.0249	98.11	94.19	3.84	84.93
D2	24320566	23906190	0.0256	97.81	93.51	4.29	86.44
D1	24932274	24433278	0.0259	97.7	93.29	6.68	84.32

^*^Percentage of bases with quality score ≥20 (bases with accuracy of 99%). ^**^Percentage of bases with quality score ≥30 (bases with accuracy of 99.9%).

### 3.7 GO and KEGG analysis of DEGs

**Figure 4:**
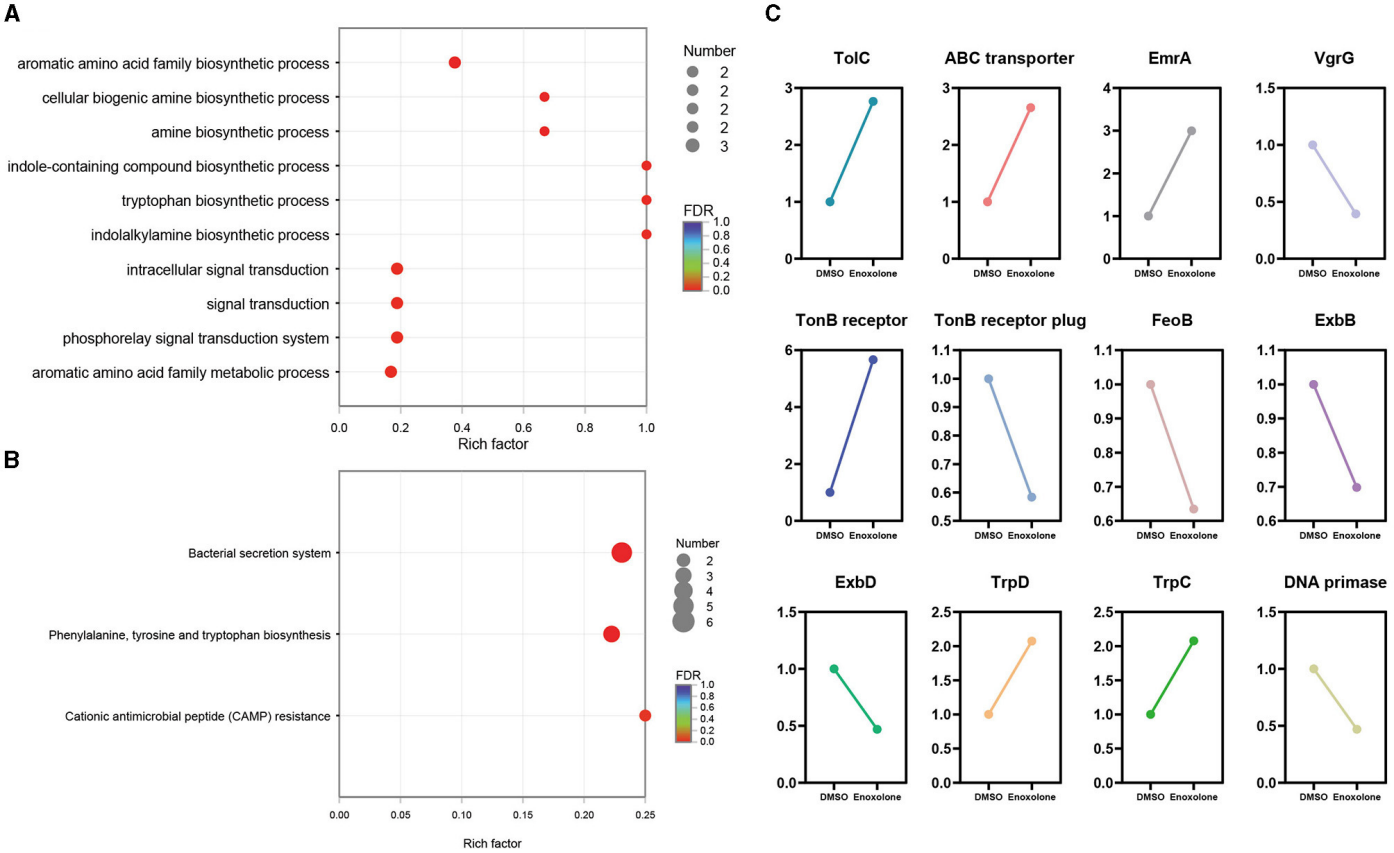
The expression pattern of genes in Enoxolone treatment. **(A)** Gene Ontology (GO) enrichment analysis of differentially expressed genes (DEGs); **(B)** enrichment of the KEGG pathway in different groups. The vertical axis shows the pathway name, the horizontal axis shows the rich factor corresponding to the pathway, and the magnitude of the FDR-value is indicated by the color of the dots. The smaller the FDR-value is, the closer the color is to red. The number of differentially expressed genes in each pathway is indicated by the size of the dots. **(C)** Expression pattern of the selected genes.

GO and KEGG annotation were performed to further understand the biological functions of the DEGs. Supplementary Figure S13A shows the annotation of the top 10 terms of the DEGs in the GO database. The annotated results showed that the most significant changes in the expression of genes related to integral components of the membrane were observed after Enoxolone treatment. In addition, there were differences in the expression of genes associated with the DNA binding, plasma membrane, and phosphorelay signal transduction systems. KEGG annotation showed that genes related to membrane transport and amino acid metabolism showed the most significant changes in expression after Enoxolone treatment (Supplementary Figure S13B). The GO and KEGG pathway enrichment analyses were conducted by scripting with the R language, and the pathways were considered to be significantly enriched when *p* < 0.05. The GO analysis indicated that the main differently expressed pathways in the Enoxolone-treated *F. columnare* included amino acid anabolism, biogenic amine synthesis, indole-containing compound synthesis, and signal transduction (Figure 4A). The bacterial secretion system, phenylalanine, tyrosine and tryptophan biosynthesis, and cationic antimicrobial peptide (CAMP) resistance were the main enriched KEGG pathways (Figure 4B). Due to the limited number of pathways in the database for the enrichment analysis, DEGs were further analyzed on a case-by-case basis. The analysis showed that the TonB-dependent siderophore receptor and the biopolymer transporter *ExbD* were labeled as up- and down-regulating DEGs, respectively. In addition, the expression of genes related to drug efflux (*TolC*, ABC transporter, *EmrA*) and amino acid synthesis (*TrpD, TrpC*) was significantly up-regulated; in contrast, the expression of genes related to iron ion transport (TonB receptor plug, *ExbD, ExbB, FeoB*), DNA synthesis (DNA primase), and the type VI secretion system (*VgrG*) was significantly down-regulated (Figure 4C). Notably, the TonB-dependent siderophore receptor is expressed in the opposite trend to its plug structure. The down-regulation of the expression of the plug structure as a binding site may indicate that the negative feedback regulation of the strain is due to the inhibition of the activity of the TonB-dependent siderophore receptor upon binding to Enoxolone.

### 3.8 Determination of the anti-*F. columnare* mechanism of Enoxolone

**Figure 5:**
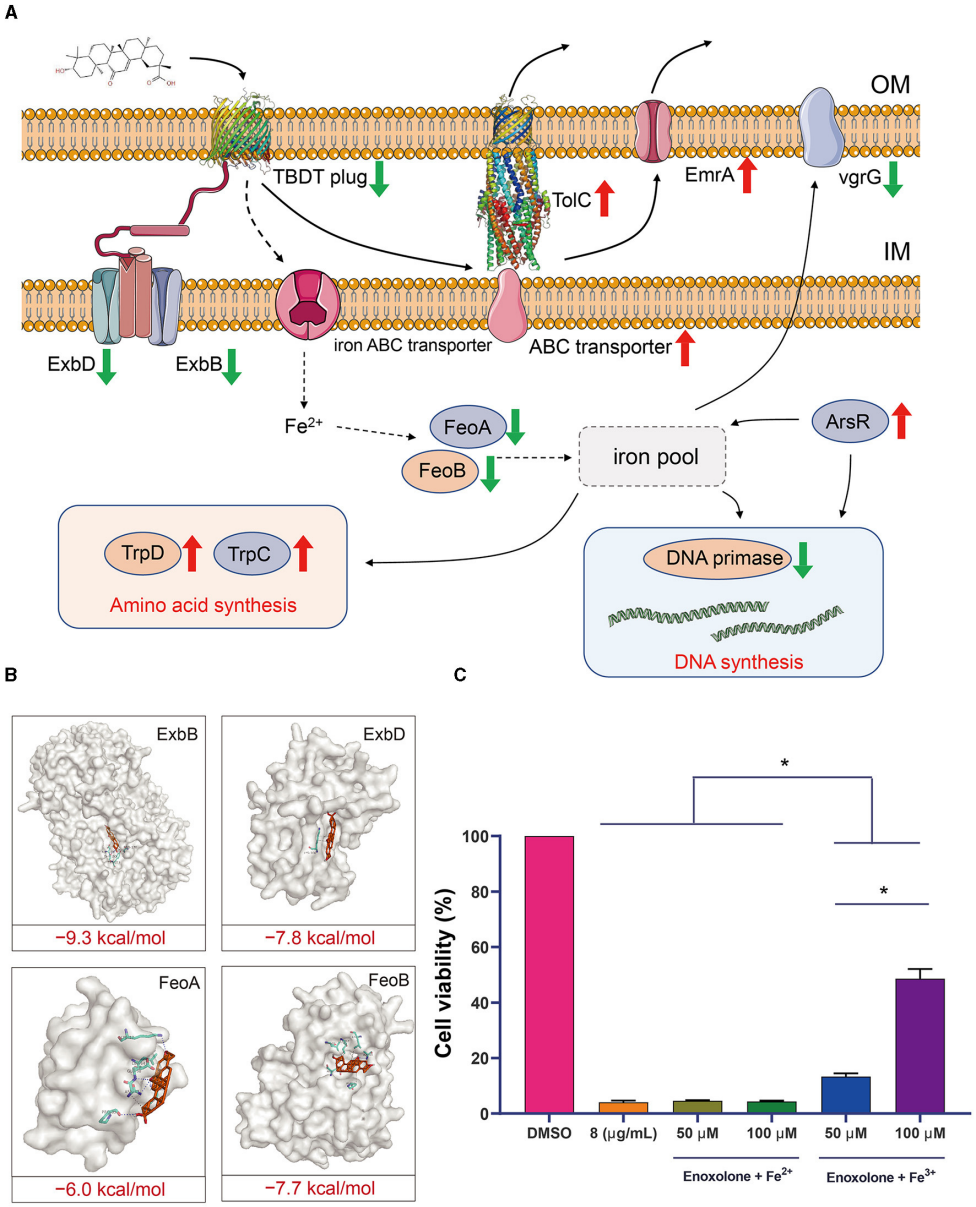
Speculation on the mechanism of inhibition of *Flavobacterium columnare* by Enoxolone. **(A)** Response network of *Flavobacterium columnare* to Enoxolone treatment mapped based on transcriptomic data. The red and green arrows represent a significant increase and decrease in gene expression, respectively. **(B)** Molecular docking results of proteins with significantly reduced expression other than TonB-dependent siderophore receptor in the TonB-dependent iron uptake system with Enoxolone. The blue and gray dashed lines represent hydrogen bonding and hydrophobic interactions, respectively. **(C)** Effect of exogenous addition of iron ions on the viability of *Flavobacterium columnare* after Enoxolone treatment. *p* ≤ 0.05 is considered significant. The error bars indicate the means ± standard deviation. *P < 0.05.

The gene response network of *F. columnare* following Enoxolone treatment was mapped against the screened genes to clarify the mechanism of inhibition. The results showed that the TonB-dependent iron transport pathway was inhibited after Enoxolone treatment, and that Enoxolone entering the bacterial cytosol via the TonB-dependent siderophore receptor activated the drug efflux system and the amino acid synthesis pathway. In addition, the relative decrease in iron ion content in bacteria inhibited DNA synthesis and reduced bacterial virulence (Figure 5A). According to the mapping of this gene regulation network, we hypothesized that the mechanism of Enoxolone resistance in *F. columnare* may be related to its competitive binding to the TonB-dependent siderophore receptor, resulting in an inadequate uptake of iron ions by bacteria. To demonstrate the accuracy of the proposed hypothesis, we first performed molecular docking with Enoxolone for other proteins in the TonB-dependent iron transport pathway that were significantly down-regulated. The results showed that the binding score of these proteins to Enoxolone was as low as −9.3 kcal/mol, which is less stable than the binding of the TonB-dependent siderophore receptor to Enoxolone (Figure 5B). Subsequently, we added FeCl_3_ and FeSO_4_ after the Enoxolone treatment of *F. columnare* and assayed the cell viability with CCK-8. The results showed that the addition of FeSO_4_ had no significant effect on cell viability, while the addition of FeCl_3_ significantly increased cell viability in a concentration-dependent manner (Figure 5C). Therefore, competitive binding to TonB-dependent siderophore receptors, leading to the inadequate uptake of iron ions, can be considered as a mechanism of Enoxolone against *F. columnare*. At the same time, the results based on the prokaryotic transcriptome also demonstrate the accuracy of the molecular docking virtual screening.

### 3.9 Biotoxicity of Enoxolone to largemouth bass

**Figure 6:**
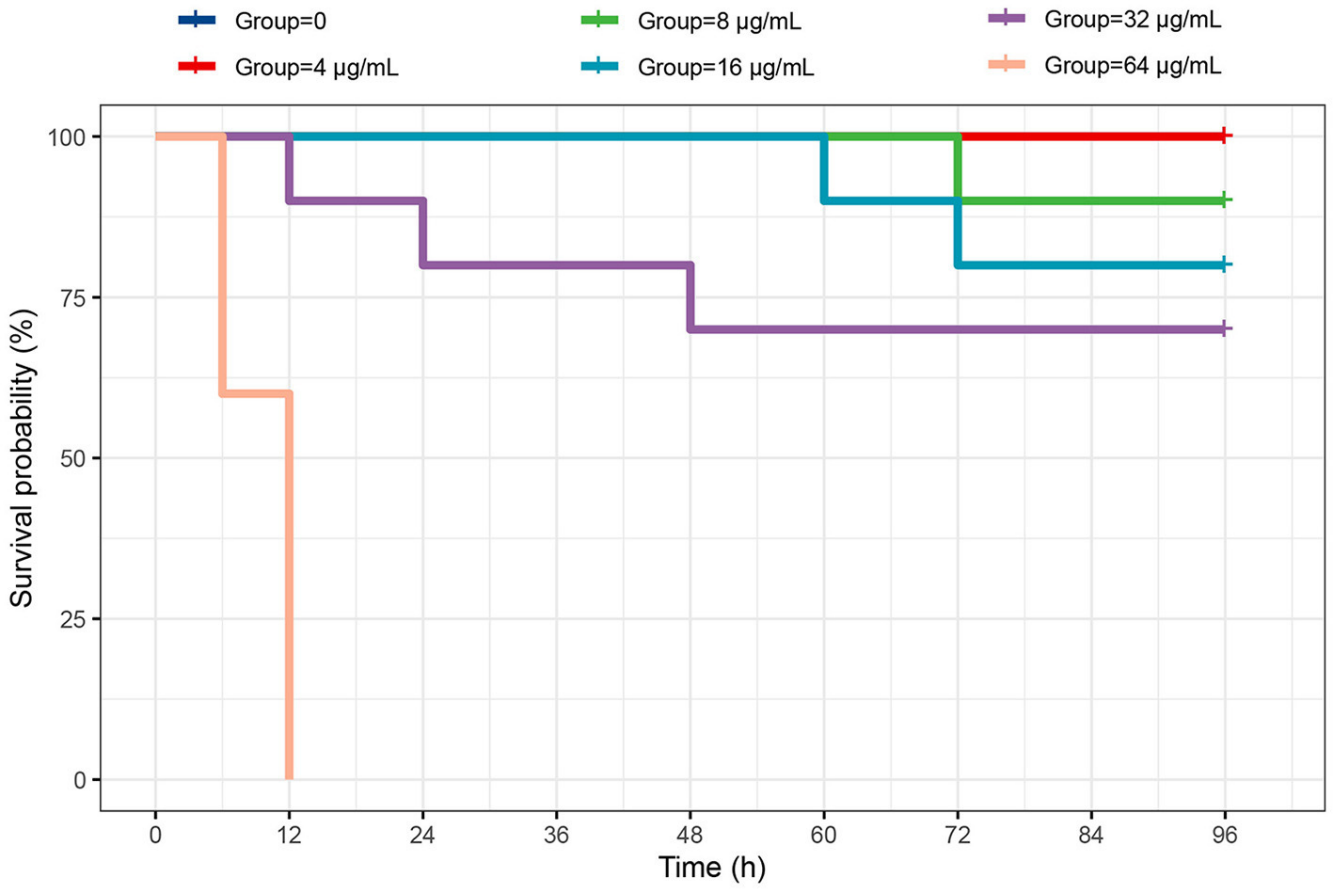
Survival rate (%) of largemouth basses immersed in different concentrations of Enoxolone.

To evaluate the toxicity, largemouth bass were selected as experimental animals and exposed to Enoxolone (0–64 μg/mL). No mortality was observed at a lower concentration (4 μg/mL). However, this increased in a concentration-dependent manner. Compared to the control group, the group treated with 64 μg/mL of Enoxolone showed 100% mortality at 12 h. Moreover, only one mortality was observed in the minimal inhibitory concentration of Enoxolone tested (8 μg/mL) compared to that in the control group during the observation period (Figure 6). The biological toxicity exhibited by Enoxolone is acceptable, considering the small size of the experimental fish used and the availability of short and spaced treatments for production applications.

## 4 Discussion

An important objective in the initial stages of drug development is to evaluate lead compounds for their potential interactions with target proteins and pharmacological activity. Additionally, there is a continuous effort to enhance the effectiveness and safety of these compounds. Due to this reason, the pharmaceutical industry has often used the experimental screening of large libraries of chemicals against targets as a traditional method of identifying novel lead compounds. Due to the swift progress in computer hardware, software, and algorithms, virtual screening techniques in drug discovery and development provide the benefits of enhanced speed, cost-effectiveness, and efficiency compared to conventional methods. In this study, the TonB-dependent siderophore receptor of *F. columnare* was selected as the target protein for a molecular docking-based virtual screening. Through the integration of laboratory research, we successfully obtained Enoxolone, a pentacyclic triterpenoid that exhibits superior bacterial suppression and precise targeting of the target protein.

Prior to virtual screening, it is often necessary to create a ligand library to hold the compounds used for screening. Currently, the most commonly used databases for the virtual screening of compounds are ZINC, Pubchem, and Drugbank, which contain the structures of millions of compounds. Nevertheless, the majority of the compounds in these databases are employed in the process of creating pharmaceuticals intended for human consumption. Mixing human and fishery medicines will raises biosecurity concerns. Nevertheless, there is currently no existing database specifically designed for screening aquatic drugs. In recent years, the effective components from TCM have exhibited promising applications in the prevention and control of fish diseases. Several studies have shown that the effective components in TCM used in aquaculture have powerful antiviral, antibacterial, antiparasitic, and antifungal effects, as well as activating the immune system in fish (Zhang et al., 2022). Furthermore, TCM possesses the benefits of abundant resources, affordability, and non-toxicity, making it highly compatible with the requirements for fishery medicine application (Zhu, 2020). Therefore, it is feasible and promising to screen efficient active ingredients and monomers with anti-pathogenic microbial activity from TCM.

In the results of the final virtual screening, we found that the pentacyclic triterpenoids in the compound library generally scored well. Pentacyclic triterpenoids have a wide range of biological activities and are commonly found as secondary metabolites in plants. Studies have shown that pentacyclic triterpenoids have anticancer, antitumor, and antiviral activities (Martinez et al., 2013). In addition, pentacyclic triterpenoids have broad-spectrum antibacterial activity against both Gram-positive and Gram-negative bacteria (Tabopda et al., 2009; Wu et al., 2021). Generally, the antibacterial activity of pentacyclic triterpenoids is thought to be related to changes in the structure and function of bacterial cellular structures (cell membranes, adhesins), cell morphology, gene expression, and processes such as adhesion and biofilm formation (Sycz et al., 2022). For example, Asiatic Acid ruptures the plasma membrane of *Clostridium difficile* and alters its membrane permeability (Harnvoravongchai et al., 2018). Pentacyclic triterpene derivatives possessing polyhydroxyl ring A inhibit Gram-positive bacteria growth by regulating the expression of genes associated with peptidoglycan metabolism, respiratory metabolism, and virulence (Huang et al., 2015). In addition, glycyrrhizic acid (GRA), ursolic acid (UA), and betulinic acid (BA) were shown to inhibit *Acinetobacter baumannii* biofilm formation by interfering with N-acyl homoserine lactone (ASL)-based signaling (Paul Bhattacharya et al., 2020). Therefore, pentacyclic triterpenoids have promising applications in the development of aquatic drugs, especially antibacterial drugs.

Virtual screening-based on molecular docking also has many drawbacks and limitations. Furthermore, computational outcomes cannot serve as a substitute for experimental data and must be combined with other methods. To further identify the lead compounds, we performed MIC assays on the top-scoring compounds. Ultimately, we isolated Enoxolone, which exhibited the most potent antibacterial activity. As one of the main active substances in *Glycyrrhiza uralensis* Fisch, Enoxolone has been reported to play a role in the treatment of hyperlipoidemia and cholestasis (Eu et al., 2010; Wang et al., 2017). In addition to the treatment of liver disease, Enoxolone has also been reported to have a wide range of antibacterial activity. Studies have shown that Enoxolone and its derivatives have good antibacterial activity against Gram-positive bacteria, including *Bacillus subtilis* and *Staphylococcus aureus*. In addition, Enoxolone can also be used in combination with other antibiotics to increase the antibacterial activity of the antibiotic against Gram-positive bacteria (de Breij et al., 2016). Antimicrobial studies of Enoxolone against Gram-negative bacteria have mostly focused on pathogens that are infectious to humans, such as *Escherichia coli* and *Neisseria gonorrhea* (Kong et al., 2018; Zhao and Su, 2023). Furthermore, licorice and its extracts have demonstrated the ability to enhance growth and immunological parameters in aquaculture. This is due to their secure and natural botanical origin, as well as their wide range of biological activities (Abdel-Tawwab and El-Araby, 2021). In this study, Enoxolone was found to have significant antibacterial activity against *F. columnare*, which extends the antibacterial spectrum of Enoxolone and provides novel insights for the design of new antibacterial agents for aquatic applications.

At present, the specific mechanism of bacterial inhibition by Enoxolone has not been described systematically. Studies on *Xanthomonas oryzae pv. oryzae* and SiHa cells have shown that Enoxolone and its derivatives can cause excessive production and accumulation of intracellular ROS and thus induce apoptosis (Lee et al., 2008; Song et al., 2022). Enoxolone may also exhibit antibacterial activity against *Staphylococcus aureus* by affecting carbohydrate and amino acid metabolism (Oyama et al., 2016). Bioinformatics calculations show that histone-like DNA-binding proteins are potential drug targets for Enoxolone against *Helicobacter pylori* (Raj et al., 2020). Deep learning-based predictions show that glycyrrhetinic acid exhibits a high affinity for ABC transporter proteins (Alkhadrawi et al., 2022). At later stages of antibiotic treatment, widespread downstream gene expression interferes with the determination of the actual binding target and antimicrobial mechanism. In this study, the bacterial transcriptome profile was analyzed immediately after 2 h of Enoxolone treatment in order to clarify the inhibition mechanism of Enoxolone against *F. columnare*. During validation trials, although the addition of divalent iron ions at high concentrations may have resulted in a toxic Fenton reaction, the addition of trivalent iron ions significantly reduced the bacteriostatic activity of Enoxolone. The final results suggest that the competitive binding of Enoxolone to the TonB-dependent siderophore receptor leads to an insufficient uptake of iron ions as a potential inhibitory mechanism against *F. columnare*. The distinctive inhibitory action of Enoxolone enables its potential advancement as a specific antibacterial agent against *F. columnare*.

While significant antibacterial activity was observed, Enoxolone also exhibited some biotoxicity. Although this biotoxicity is acceptable for the purpose of bacterial inhibition, it is still necessary to reduce the toxicity to accommodate high-concentration applications. In fact, further structural modifications and treatments are often required after lead compounds are obtained. Research has demonstrated that the combination of Enoxolone and emodin esters enhances the hatching and survival rates of zebrafish embryos. Additionally, it decreases the occurrence of cardiomyocyte malformation and apoptosis (Zhong et al., 2022). In addition, Enoxolone derivatives obtained via reduction at C-11, oxidation at C-3, and condensation at C-2 exhibited stronger anti-staphylococcal activity than Enoxolone (Yang et al., 2020). Therefore, the modification of the structure to further improve its efficacy and reduce biotoxicity could be the next step in the study of Enoxolone for the treatment of *F. columnare* infection.

## 5 Conclusion

In conclusion, we obtained Enoxolone with anti-*F. columnare* activity from traditional Chinese medicine compounds using a virtual screening technique. Moreover, we demonstrated through laboratory experiments that Enoxolone targets the TonB-dependent siderophore receptor of *F. columnare* and exhibits an acceptable biological toxicity. Along with obtaining antimicrobial lead compounds, our results also declare the usability of virtual screening techniques in the development of novel antimicrobial agents for bacteria infecting aquatic organisms.

## Data availability statement

The datasets presented in this study can be found in online repositories. The names of the repository/repositories and accession number(s) can be found below: https://www.ncbi.nlm.nih.gov/, PRJNA971873.

## Ethics statement

The animal study was approved by the Animal Care and Use Committee of Sichuan Agricultural University. The study was conducted in accordance with the local legislation and institutional requirements.

## Author contributions

ML: Data curation, Formal analysis, Writing—original draft. BC: Data curation, Formal analysis, Writing—review & editing. MX: Data curation, Formal analysis, Writing—review & editing. FL: Data curation, Writing—review & editing. YG: Conceptualization, Writing—review & editing. DC: Investigation, Visualization, Writing—review & editing. PO: Methodology, Writing—review & editing. XH: Conceptualization, Supervision, Writing—review & editing. YD: Supervision, Writing—review & editing.
