# Supplementary material for: Identification of TonB-dependent siderophore receptor inhibitors against Flavobacterium columnare using a structure-based high-throughput virtual screening method
Source: Front Microbiol. 2024 May 21;15:1392178. doi: 10.3389/fmicb.2024.1392178 (PMC11148330; doi:10.3389/fmicb.2024.1392178)
Supplement: Supplementary file 1 [file Data_Sheet_1.zip › Supplementary File.docx]

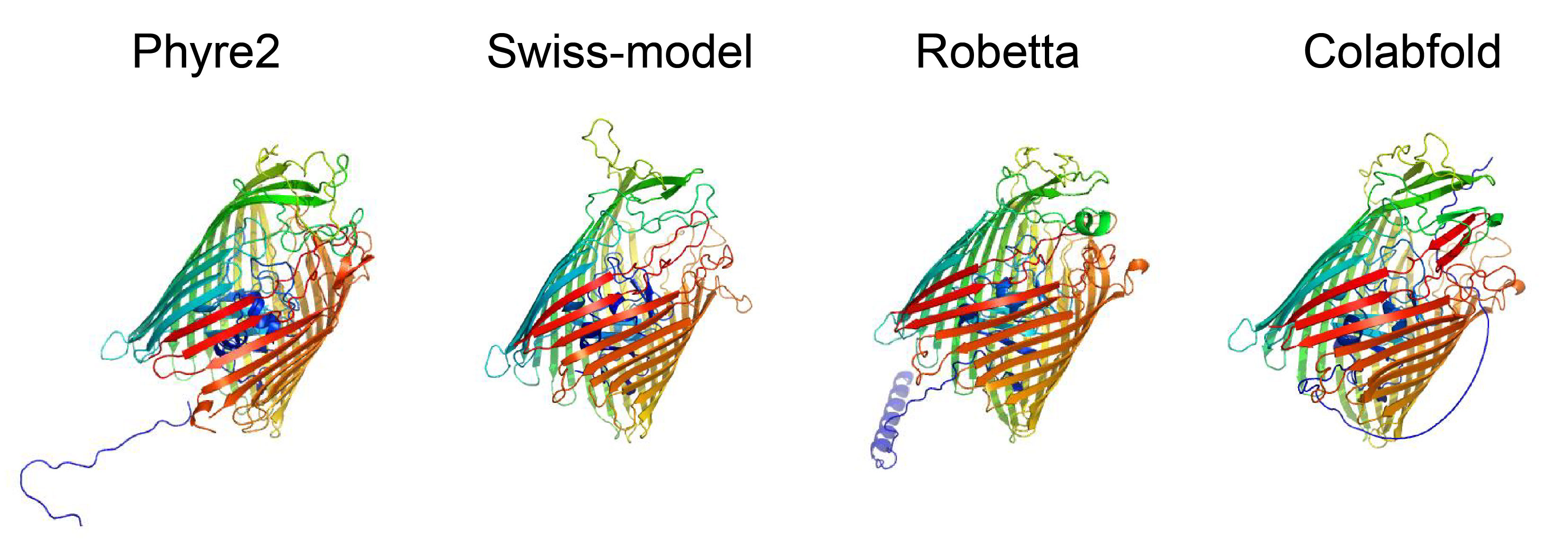


**Figure S1. Three-dimensional structures of TonB-dependent siderophore receptor of *Flavobacterium columnare* obtained by different homology modeling approaches.**


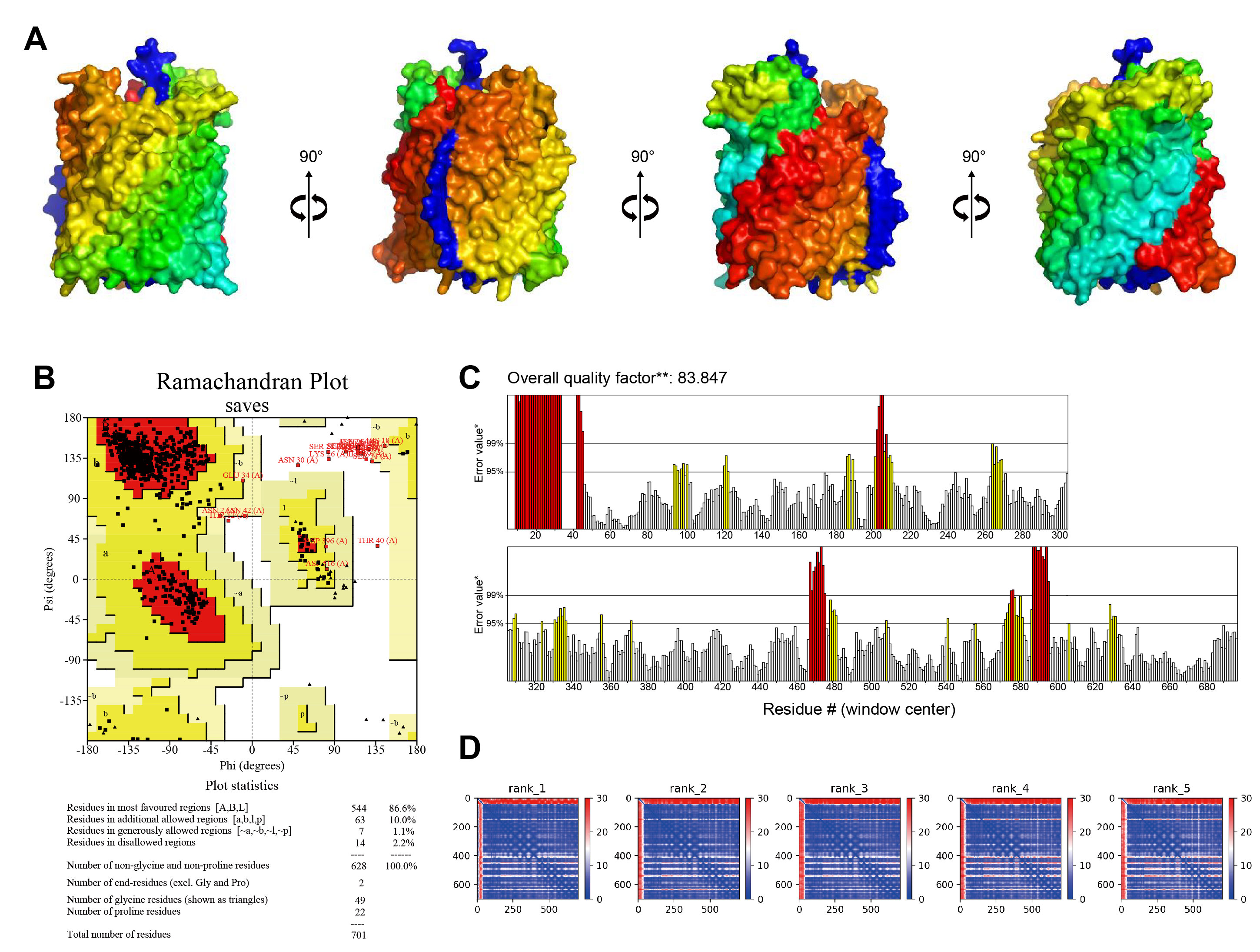


**Figure S2. Validation of a three-dimensional structural model of the TonB-dependent siderophore receptor of Flavobacterium columnare constructed by ColabFold.** A: Color-coded surface structure of the TonB-dependent siderophore receptor of *Flavobacterium columnare*; B: All atom presentation of TonB-dependent siderophore receptor protein model in Ramachandran plot by PROCHECK server; C: Assessment of TonB-dependent siderophore receptor protein amino acid resolution by the ERRAT2 program; D: ColabFold construction model for amino acid prediction alignment error assessment. *On the error axis, two lines are drawn to indicate the confidence with which it is possible to reject regions that exceed that error value.


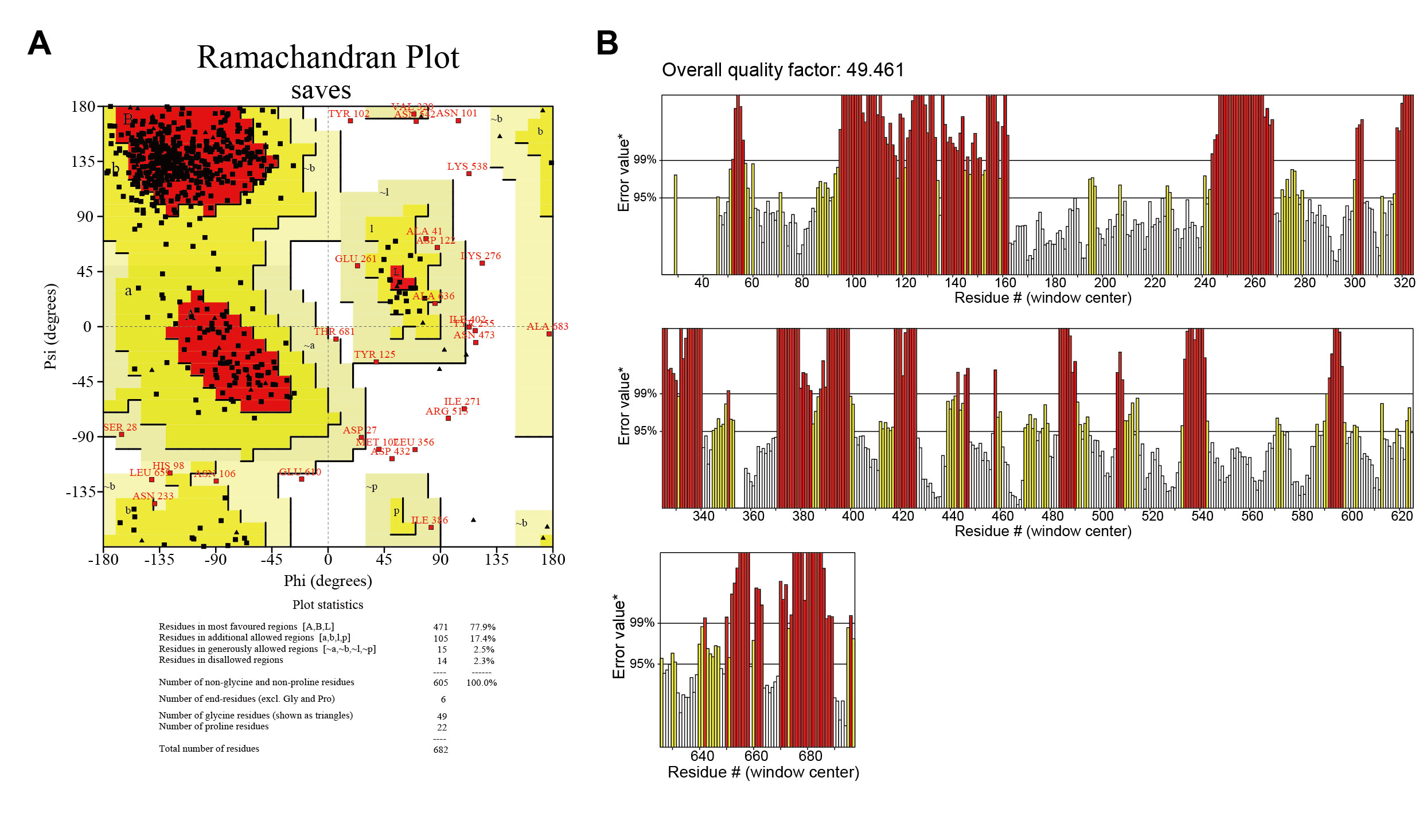


**Figure S3. Validation of a three-dimensional structural model of the TonB-dependent siderophore receptor of *Flavobacterium columnare* constructed by Phyre2.** A: All atom presentation of TonB-dependent siderophore receptor protein model in Ramachandran plot by PROCHECK server; B: Assessment of TonB-dependent siderophore receptor protein amino acid resolution by the ERRAT2 program. *On the error axis, two lines are drawn to indicate the confidence with which it is possible to reject regions that exceed that error value.


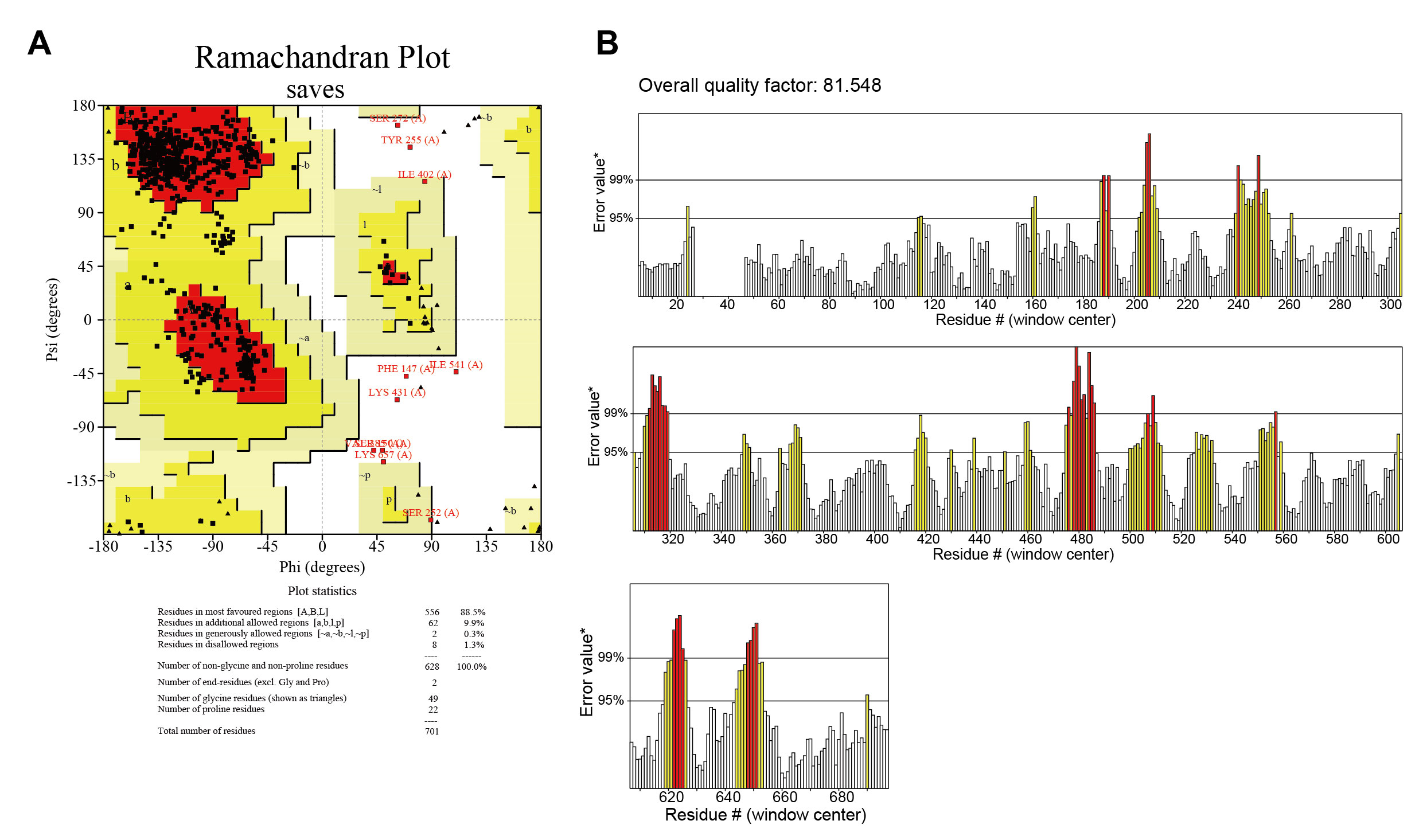


**Figure S4. Validation of a three-dimensional structural model of the TonB-dependent siderophore receptor of *Flavobacterium columnare* constructed by Robetta.** A: All atom presentation of TonB-dependent siderophore receptor protein model in Ramachandran plot by PROCHECK server; B: Assessment of TonB-dependent siderophore receptor protein amino acid resolution by the ERRAT2 program. *On the error axis, two lines are drawn to indicate the confidence with which it is possible to reject regions that exceed that error value.


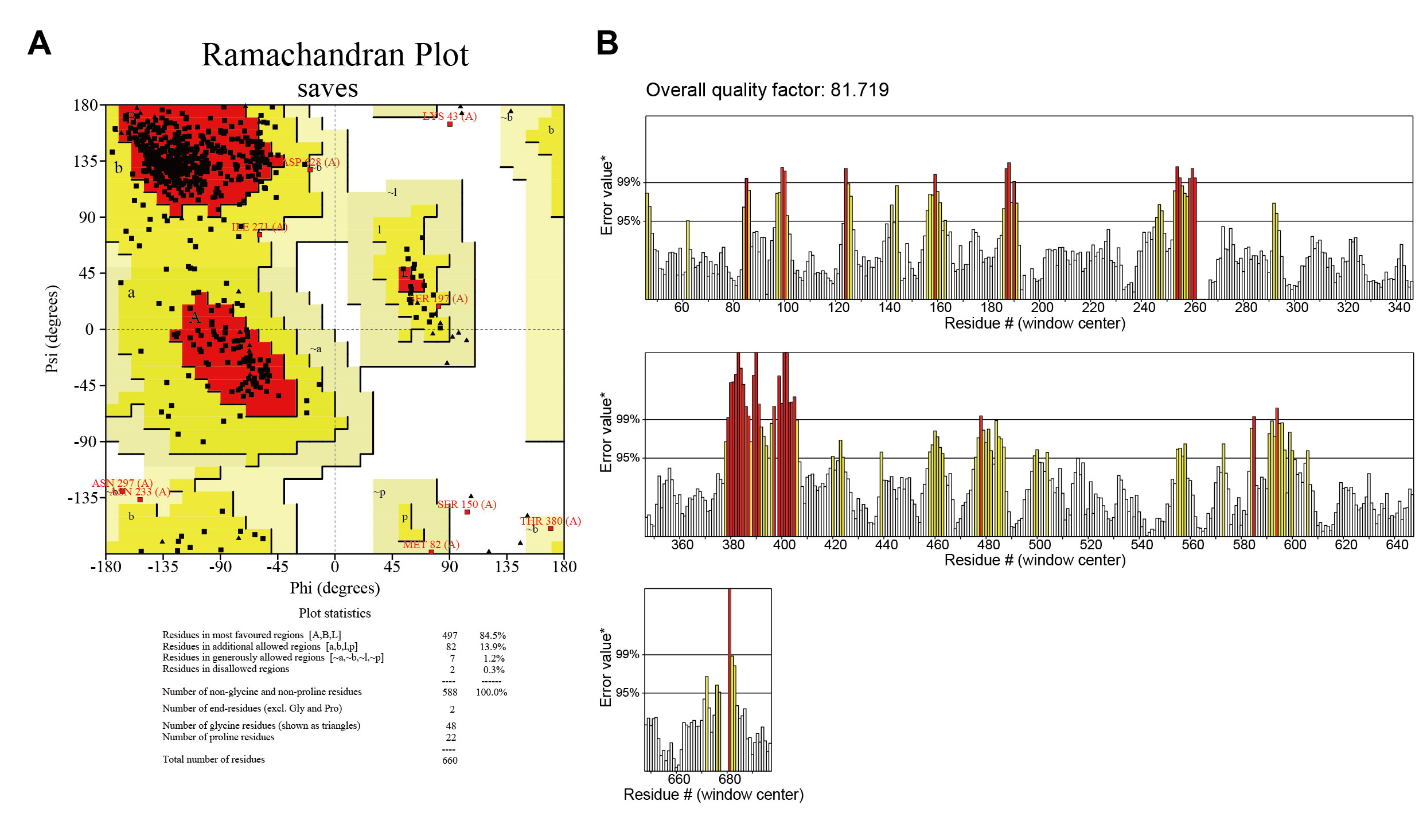


**Figure S5. Validation of a three-dimensional structural model of the TonB-dependent siderophore receptor of *Flavobacterium columnare* constructed by SWISS-MODEL.** A: All atom presentation of TonB-dependent siderophore receptor protein model in Ramachandran plot by PROCHECK server; B: Assessment of TonB-dependent siderophore receptor protein amino acid resolution by the ERRAT2 program. *On the error axis, two lines are drawn to indicate the confidence with which it is possible to reject regions that exceed that error value.


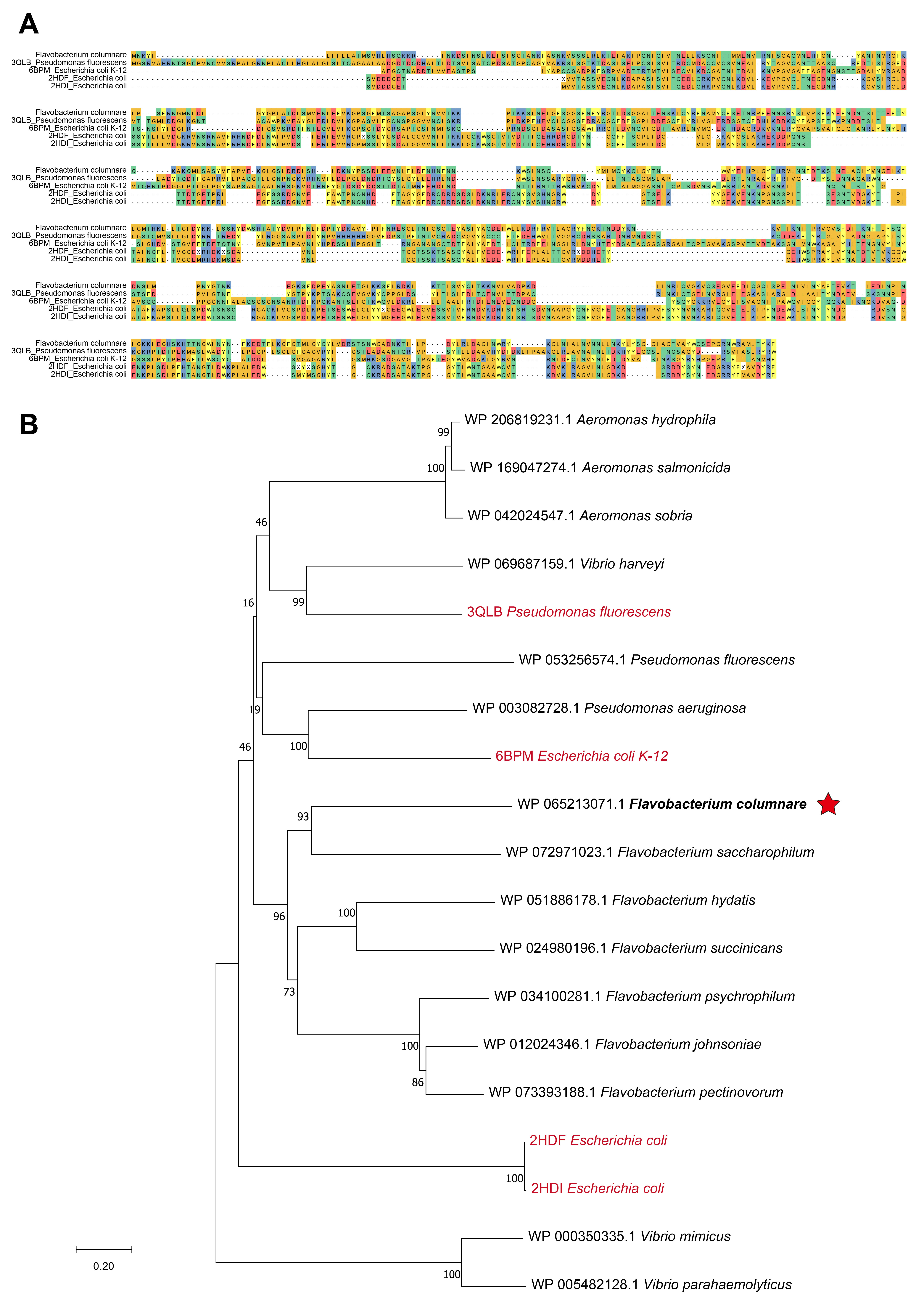


**Figure S6. Multiple sequence comparison and evolutionary relationships of TonB-dependent siderophore receptor.** A: Multiple sequence comparison of target proteins with crystal-structured proteins obtained from BLASTp against PDB database (Identity: 3QLB 24.82%, 6BPM 21.51%, 2HDF 22.22%, 2HDI 22.22%). B: Phylogenetic tree of target proteins and proteins from the PDB database (red) with common aquatic pathogen proteins.


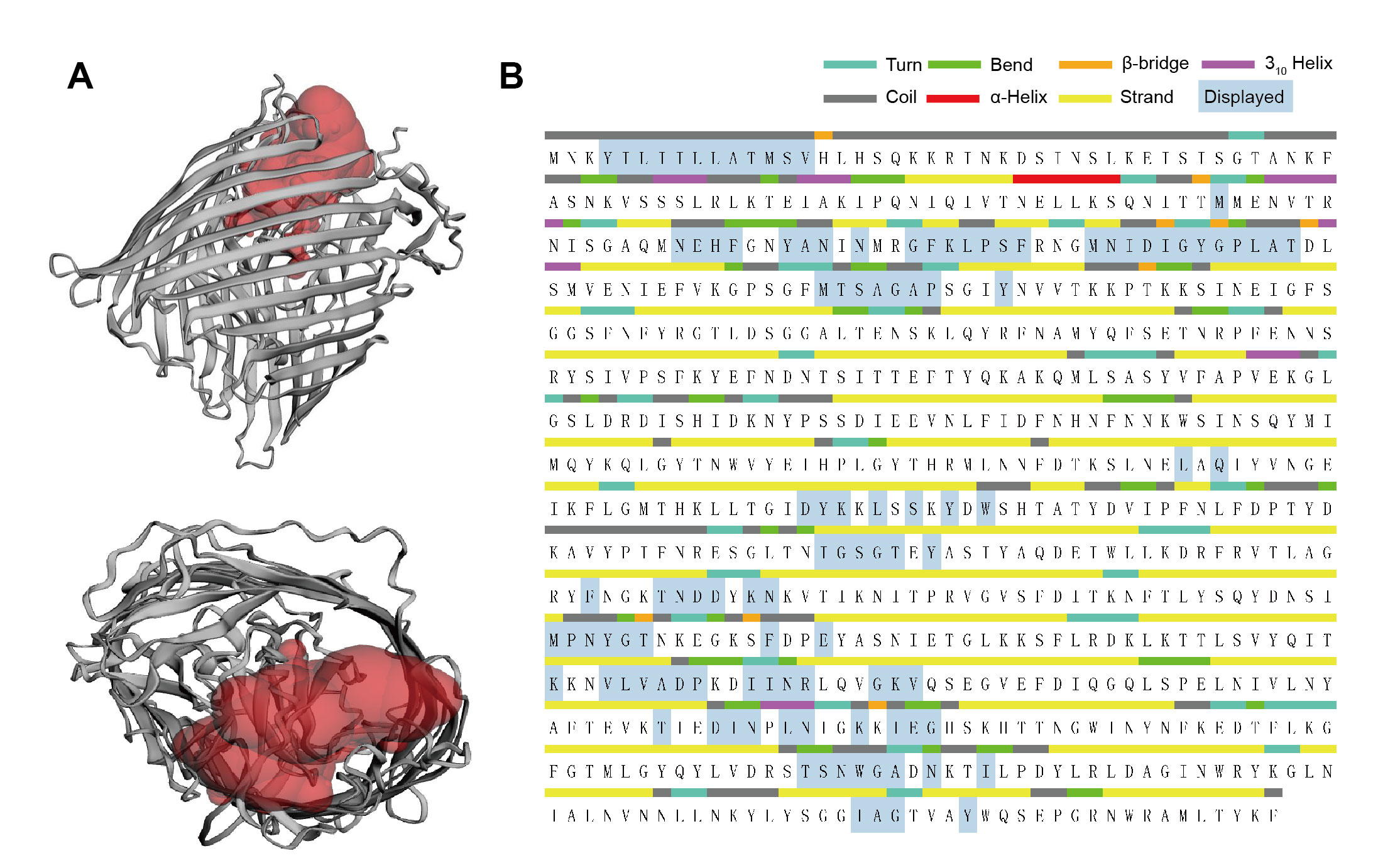


**Figure S7. CASTp predicts TonB-dependent siderophore receptor active sites in *Flavobacterium columnare*.** A: Biological structure and binding sites of TonB-dependent siderophore receptor detected by the CASTp server; B: Panel of amino acid sequences and annotations obtained by CASTp analysis.


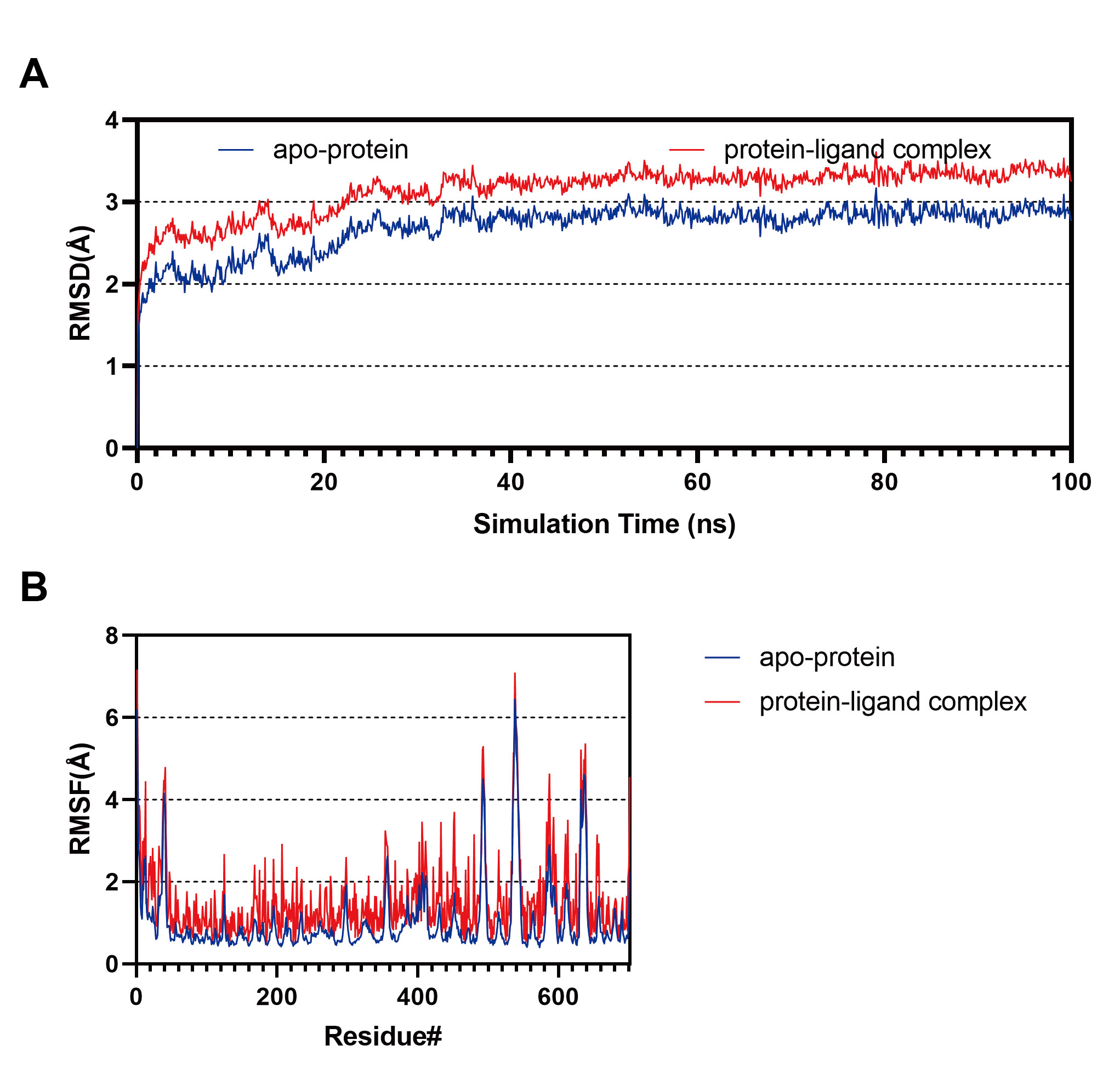


**Figure S8. Comparison of RMSD (A) and RMSF (B) for apo-protein and protein-ligand complex.**


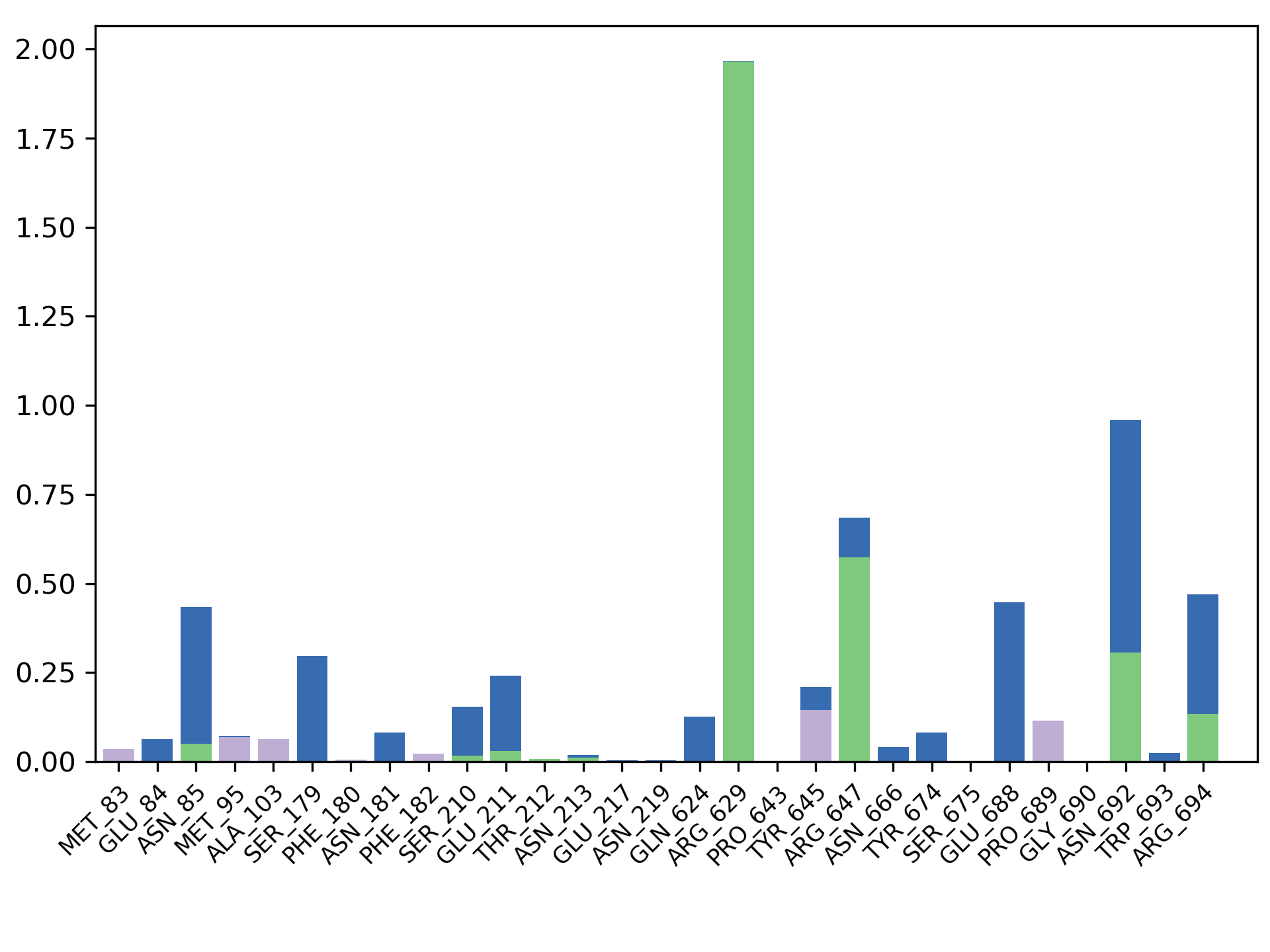


**Figure S9. Normalized stacked bar chart representation of relatively stable receptor-ligand interactions over the course of the MD trajectories.** Green, purple and blue colors represent hydrogen bonds, hydrophobic interactions and water bridges, respectively.


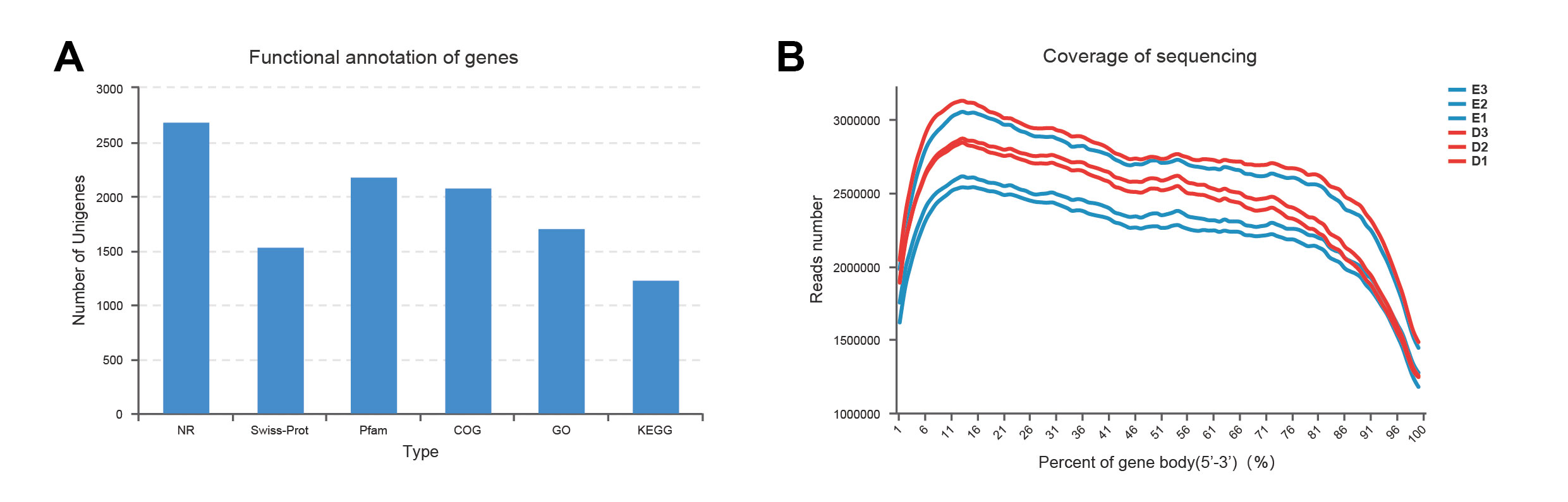


**Figure S10. Sequencing coverage distribution of transcriptome.** The horizontal coordinate is the percentage of base length of a single gene to the total base length; the vertical coordinate is the sum of the sequence entries in the corresponding interval at the position of the horizontal axis compared to all genes.


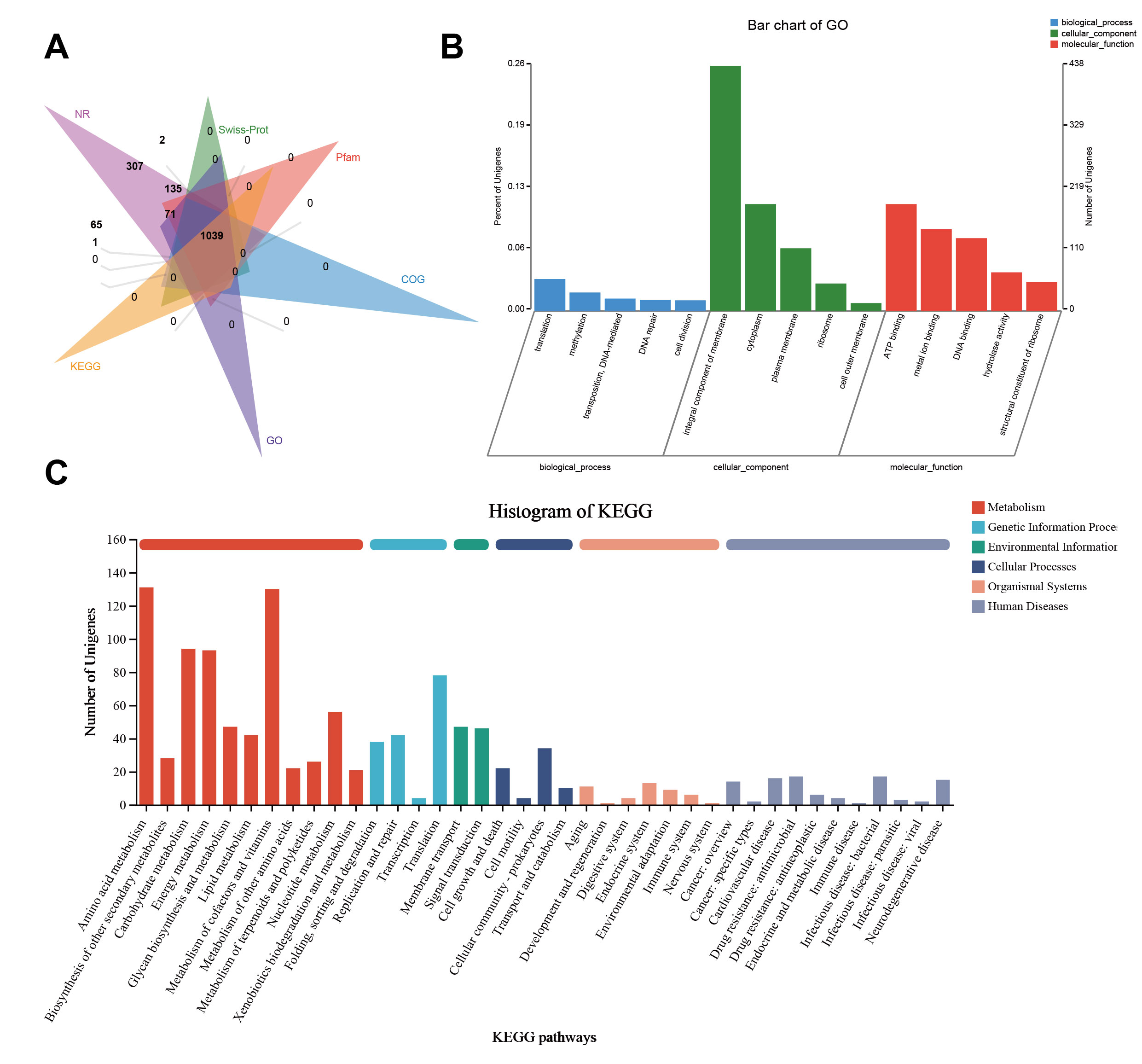


**Figure S11. Database annotation of transcriptome.** A: Numbers of annotated unigenes in public databases; B: Gene ontology (GO) annotations of the assembled unigenes. Each annotated sequence is assigned at least one GO term of the following: biological process, cellular component, or molecular function; C: Cluster of KEGG Ortholog database annotations of assembled unigenes.


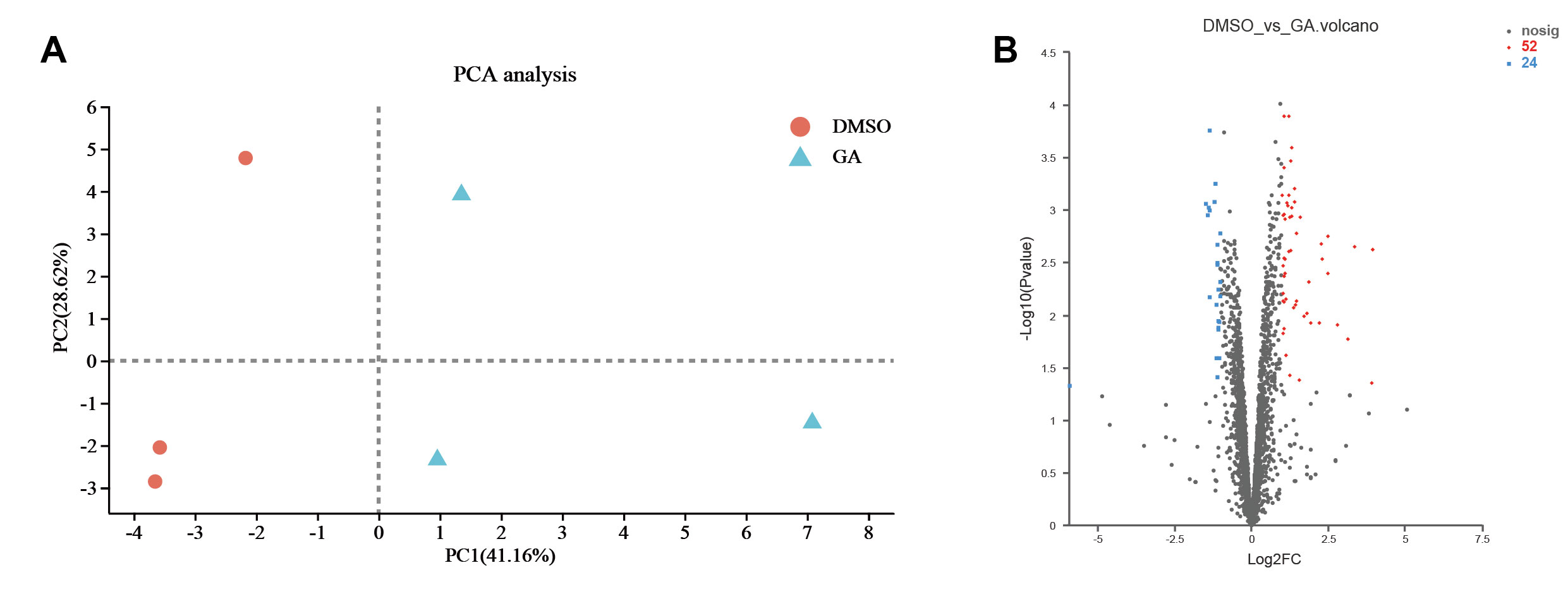


**Figure S12. Differential analysis of transcriptome between control and Enoxolone treatment group.** A: Principal component analysis (PCA) between control and treatment samples. Blue triangles indicate samples from the Enoxolone treatment group, while red circles indicate samples from the control group. B: Volcano plot showing the distributions of the differentially expressed genes (DEGs). Each dot represents a gene. Red and blue dots represent up- and down regulated DEGs, respectively.


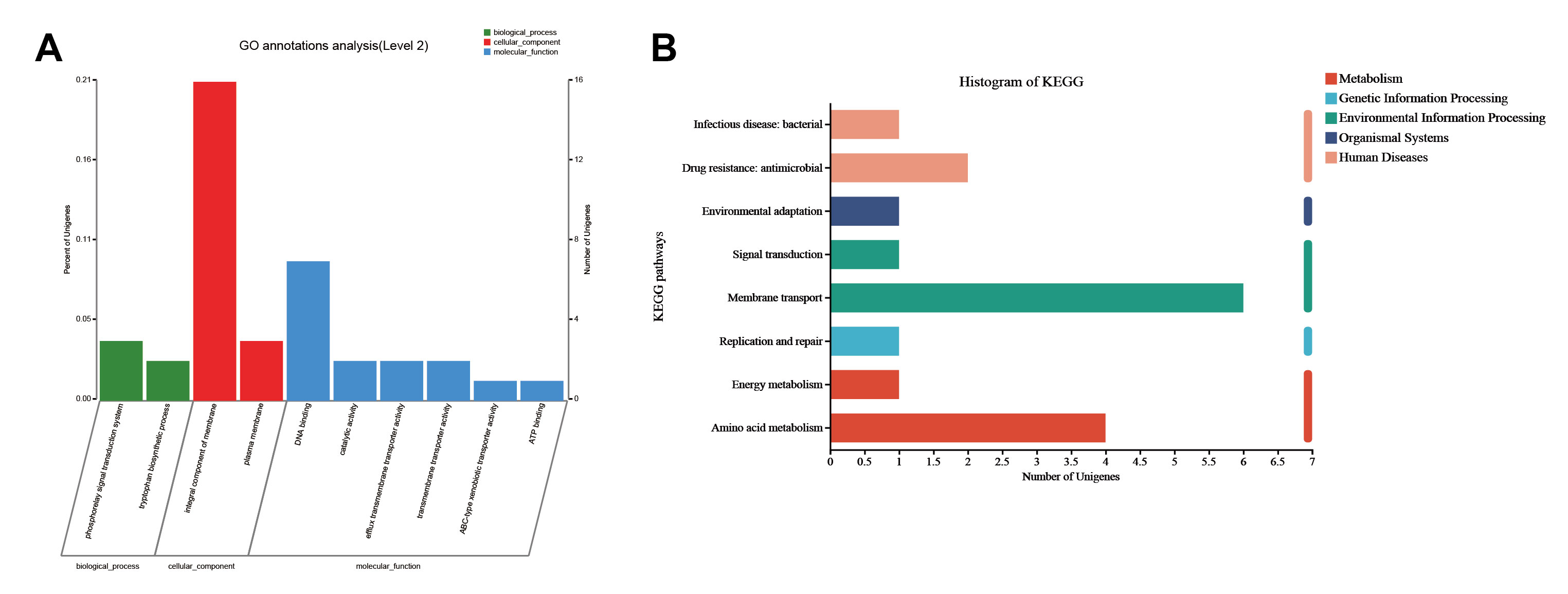


**Figure S13. Database annotation of differentially expressed genes.** A: Gene ontology (GO) database annotation of differentially expressed genes; B: KEGG database annotation of differentially expressed genes.
